# An immune-sympathetic neuron communication axis guides adipose tissue browning in cancer-associated cachexia

**DOI:** 10.1073/pnas.2112840119

**Published:** 2022-02-24

**Authors:** Hao Xie, Christoph Heier, Xia Meng, Latifa Bakiri, Isabella Pototschnig, Zhiyuan Tang, Silvia Schauer, Vanessa J. Baumgartner, Gernot F. Grabner, Gernot Schabbauer, Heimo Wolinski, Graham R. Robertson, Gerald Hoefler, Wenwen Zeng, Erwin F. Wagner, Martina Schweiger, Rudolf Zechner

**Affiliations:** ^a^Institute of Molecular Biosciences, University of Graz, 8010 Graz, Austria;; ^b^School of Medicine, Tsinghua University, 100190 Beijing, China;; ^c^Genes and Disease Group, Department of Laboratory Medicine, Medical University of Vienna, 1090 Vienna, Austria;; ^d^Department of Pharmacy, Affiliated Hospital of Nantong University, 226001 Nantong, China;; ^e^Diagnostic and Research Institute of Pathology, Medical University Graz, 8010 Graz, Austria;; ^f^Institute of Physiology, Medical University of Vienna, 1090 Vienna, Austria;; ^g^Lyramid Ltd, Sydney, 2000 NSW, Australia;; ^h^Genes and Disease Group, Department of Dermatology, Medical University of Vienna, 1090 Vienna, Austria;; ^i^Field of Excellence BioHealth, University of Graz, 8010 Graz, Austria;; ^j^BioTechMed–Graz, 8010 Graz, Austria

**Keywords:** cancer cachexia, macrophage, browning, immunometabolism, adipose tissue

## Abstract

Cancer-associated cachexia (CAC) is a hypermetabolic syndrome characterized by unintended weight loss due to the atrophy of adipose tissue and skeletal muscle. A phenotypic switch from white to beige adipocytes, a phenomenon called browning, accelerates CAC by increasing the dissipation of energy as heat. Addressing the mechanisms of white adipose tissue (WAT) browning in CAC, we now show that cachexigenic tumors activate type 2 immunity in cachectic WAT, generating a neuroprotective environment that increases peripheral sympathetic activity. Increased sympathetic activation, in turn, results in increased neuronal catecholamine synthesis and secretion, β-adrenergic activation of adipocytes, and induction of WAT browning. Two genetic mouse models validated this progression of events. 1) Interleukin-4 receptor deficiency impeded the alternative activation of macrophages, reduced sympathetic activity, and restrained WAT browning, and 2) reduced catecholamine synthesis in peripheral dopamine β-hydroxylase (DBH)–deficient mice prevented cancer-induced WAT browning and adipose atrophy. Targeting the intraadipose macrophage-sympathetic neuron cross-talk represents a promising therapeutic approach to ameliorate cachexia in cancer patients.

## Significance

More than a half of cancer patients suffer a complex metabolic syndrome termed cancer-associated cachexia (CAC). This disorder is characterized by unintended loss of body weight and largely reduces quality of life, effectiveness of chemotherapy, and survival of cancer patients. Here, we provide a potential mechanism underlying the metabolic reprogramming and atrophy of adipose tissue in CAC. We demonstrate that miscommunication between immune cells and sympathetic neurons in adipose tissue generates a perpetual catabolic state that leads to adipose tissue loss in cachexigenic tumor-bearing mice. Targeting the signals involved in this communication process may provide therapeutic options to treat CAC.

Cancer-associated cachexia (CAC) is an energy balance disorder causing unintended loss of body weight due to depletion of white adipose tissue (WAT) and skeletal muscle. This multiorgan and multifactorial syndrome affects up to 80% of cancer patients and is responsible for more than 20% of cancer-associated deaths (1). CAC impedes the effectiveness of anticancer therapies and drastically lowers patients’ quality of life (2).

A long list of tumor-borne, often proinflammatory factors, including interleukin-6 (IL-6) (3), parathyroid hormone–related protein (PTHrP) (4), leukemia inhibitory factor (LIF) (5), zinc α-glycoprotein (6), or growth differentiation factor-15 (GDF-15) (7), trigger CAC in mouse models. However, the signaling cascades and catabolic mechanisms that lead to adipose- and muscle tissue wasting remain insufficiently understood (8, 9). IL-6 and PTHrP are among the best studied of these “cachexokines.” Their presence or absence is decisive for the development of CAC in cancer patients and animal models (4, 10–13). Thus, treatment with neutralizing antibodies against IL-6, the IL-6 receptor, or PTHrP ameliorates CAC in various mouse models of CAC (3, 4, 14, 15).

CAC-associated WAT atrophy results from a metabolic switch toward decreased lipid synthesis and excessive degradation of lipid stores via enhanced triglyceride degradation (lipolysis) (9, 16). Induced lipolysis is observed in both humans and mice with CAC (17, 18). The absence of metabolic lipases at least partially ameliorates cachexia in murine cancer models (19). The metabolic or catabolic fates of lipolytic products, namely fatty acids (FAs) and glycerol, have not been fully clarified. These may provide energy and/or biosynthetic substrates for cancer cells to promote tumor growth or can be reesterified in WAT, creating an adenosine-triphosphate (ATP)-consuming futile metabolic cycle. Both of these pathways would contribute to the eventual loss of WAT during CAC (20).

Another important catabolic pathway in CAC involves the direct oxidation of FAs and glycerol in adipose tissue. This process is promoted by the conversion of white to beige adipocytes called “WAT browning.” During WAT browning, adipocytes adopt a multilocular lipid droplet morphology; express genes that are typical for brown adipocytes, such as uncoupling protein-1 (UCP-1); exhibit elevated substrate oxidation rates; and dissipate energy as heat (21). WAT browning occurs in carcinogen-induced cancer models and genetically engineered mouse models as well as syngeneic and xenogeneic transplant models of murine cancers (3, 4, 22) and depends on the presence of cachexokines. WAT browning also occurs in humans suffering CAC or severe burn trauma (3, 23–25), but the cellular and molecular mechanisms underlying catabolic WAT remodeling in CAC remain unclear.

Here, we demonstrate that a macrophage-sympathetic neuron signaling axis generates a high β-adrenergic tone resulting in beige adipogenesis, increased lipid degradation, and WAT atrophy in murine models of CAC. This mechanism triggering hypermetabolism in CAC may offer targets for prevention or treatment of the disease.

## Results

### CAC Is Associated with WAT Browning, but Cachexokines Do Not Directly Stimulate Thermogenic Gene Expression in Adipocytes.

**Fig. 1.**
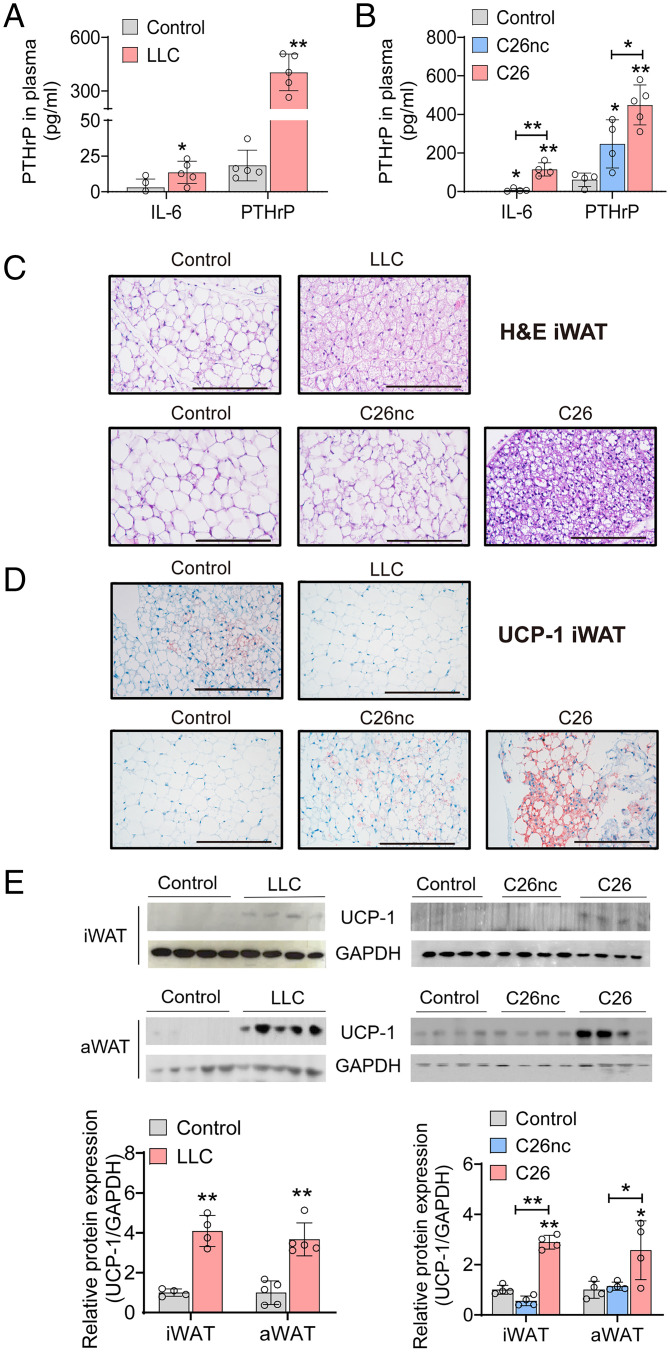
CAC is associated with increased plasma IL-6 and PTHrP concentrations and WAT browning. (*A*–*E*) Mice bearing LLC, C26, or C26nc tumors were euthanized 14 to 16 d after tumor cell inoculation. (*A* and *B*) PTHrP and IL-6 plasma concentrations were determined in LLC (*A*), C26, and C26nc (*B*) tumor-bearing mice using ELISA (*n* = 4 to 5 per group). (*C* and *D*) Representative iWAT sections of LLC, C26, and C26nc tumor-bearing mice stained with hematoxylin and eosin (H&E; *C*) or immunolabeled for UCP-1 (*D*). (Scale bars: 200 µm.) (*E*) Western blotting analysis to detect uncoupling protein-1 (UCP-1) protein expression in iWAT and aWAT of LLC, C26, and C26nc tumor-bearing mice. Glycerinaldehyde-3-phosphate-dehydrogenase (GAPDH) was used as loading control. The protein contents of UCP-1 and GAPDH were quantified by densitometric analysis. Data are presented as means ± SD. Significance was determined by unpaired two-tailed Student’s *t* test (*A* and *E*) or a one-way ANOVA followed by Tukey’s post hoc analysis. *P ≤ 0.05; **P ≤ 0.01.

We employed two established murine syngeneic allograft CAC models, Lewis lung carcinoma (LLC) (26) and colon carcinoma 26 (C26) (26), and confirmed CAC development and the occurrence of WAT browning in these models. Fourteen to 16 d after tumor cell inoculation, LLC and C26 tumor-bearing mice weighed 13.2% and 12.0% less than nontumor-bearing control animals, respectively (*SI Appendix*, Fig. S1 *A* and *B*). The weights of inguinal white adipose tissue (iWAT), anterior subcutaneous white adipose tissue (aWAT), and gonadal white adipose tissue (gWAT) as well as brown adipose tissue (BAT) were significantly lower in LLC and C26 tumor-bearing mice than in the respective controls (*SI Appendix*, Fig. S1 *C* and *D*). Both WAT and muscle loss were more pronounced in the C26 model than in the LLC model. In contrast, injection of a noncachexigenic C26 cell line (C26nc) caused no reduction in body weight or adipose tissue mass (*SI Appendix*, Fig. S1 *B* and *D*), despite a similar tumor burden. Hepatosplenomegaly was evident by significantly increased liver and spleen weights in LLC (1.2-fold higher liver and 1.8-fold higher spleen weight) and C26 (1.1-fold higher liver and 1.4-fold higher spleen weight) compared with control and C26nc tumor-bearing mice, respectively (*SI Appendix*, Fig. S1 *C* and *D*). When compared with control mice or C26nc tumor-bearing mice, LLC and C26 tumor-bearing mice exhibited drastically elevated plasma concentrations of IL-6 (4.5-fold for LLC and 48.8-fold for C26 tumor-bearing mice) and PTHrP (22-fold for LLC and 7.4-fold for C26 tumor-bearing mice), two proinflammatory cytokines that have been associated with beige adipogenesis in cachexia (3, 4) (Fig. 1 *A* and *B*). PTHrP was also elevated in C26nc mice (4.1-fold higher) compared with nontumor-bearing control mice. Concentrations of tumor necrosis factor-α (TNFα) were not increased in the plasma of LLC and C26 tumor-bearing mice compared with the respective controls (*SI Appendix*, Fig. S1*E*). Histological and immunohistochemical analyses of adipose tissues showed clear signs of browning in iWAT of LLC and C26 but not of C26nc tumor-bearing mice, with reduced adipocyte size, a multilocular lipid droplet phenotype, and increased UCP-1 protein abundance (Fig. 1 *C* and *D*). Increased UCP-1 protein amount of LLC tumor-bearing mice was verified by Western blotting analyses and revealed 4- and 3.5-fold higher UCP-1 protein abundance in iWAT and aWAT, respectively. In iWAT and aWAT of C26 tumor-bearing mice, UCP-1 protein levels were 2.9- and 3.2-fold higher, respectively, than in control mice (Fig. 1*E*). These results reinforce the notion that LLC and C26 syngeneic allografts represent valid models to study the development of WAT browning and CAC in mice.

**Fig. 2.**
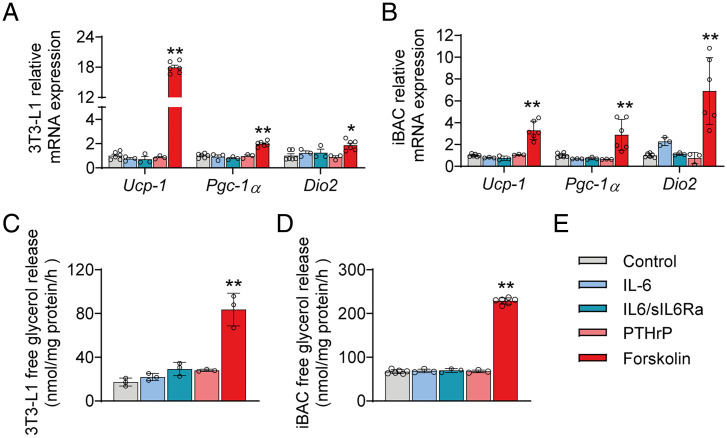
IL-6 and PTHrP do not directly stimulate thermogenic gene expression or lipolysis in white and brown adipocyte cell lines. (*A* and *B*) qRT-PCR analysis was used to detect mRNA levels of thermogenic marker genes in differentiated 3T3-L1 (white adipocytes; *A*) and iBACs (brown adipocytes; *B*) treated with 10 ng/mL PTHrP, 20 ng/mL IL-6, 20 ng/mL IL-6/37.14 ng/mL (equimolar to IL-6) sIL-6ra, 10 µM forskolin, or vehicle for 8 h. (*C* and *D*) Glycerol release from differentiated 3T3-L1 (*C*) and iBACs (*D*) treated with 40 ng/mL PTHrP, 20 ng/mL IL-6, 20 ng/mL IL-6/37.14 ng/mL (equimolar to IL-6) sIL-6ra, 10 µM forskolin, or vehicle and 2% FA-free BSA for 8 h. Data are presented as means ± SD. Significance was determined by one-way ANOVA followed by Tukey’s post hoc analysis. *P ≤ 0.05; **P ≤ 0.01. (*E*) Legend for *A–D*.

Next, we addressed the direct impact of cachexokines on WAT browning. As shown above, plasma levels of IL-6 and PTHrP are robustly elevated in LLC- and C26-treated mice but not or less so in C26nc-treated mice. To assess whether these factors are able to directly induce beige adipogenesis, we incubated differentiated 3T3-L1 cells or immortalized brown adipogenic cells (iBACs) as in vitro models for white and brown adipocytes, respectively, with recombinant PTHrP, IL-6, or the potent adenylate cyclase activator forskolin. To also account for both membrane-bound IL-6 receptor– and soluble IL-6 receptor–dependent signaling, we incubated the cells with equimolar concentrations of IL-6 and the soluble IL-6 receptor-α (sIL-6ra) (27). As expected, forskolin treatment elevated messenger ribonucleic acid (mRNA) levels of thermogenic genes via cyclic adenosine-monophosphate (cAMP) signaling including *Ucp-1* (17.5-fold), *Pgc-1α* (2-fold), and *Dio2* (1.9-fold) in 3T3-L1 cells and in iBACs by 3.9-, 4.1-, and 9.5-fold, respectively (Fig. 2 *A* and *B*). In contrast, IL-6 (20 ng/mL) or IL-6 in combination with sIL-6ra (37.14 ng/mL, equimolar to IL-6) did not increase mRNA levels of thermogenic genes in differentiated 3T3-L1 adipocytes or iBACs (Fig. 2 *A* and *B*), despite a pronounced induction of the classical janus kinase (JAK)/signal transducer and activator of transcription (STAT) signaling pathway as evident by fourfold increased STAT3 phosphorylation at Tyr705 (*SI Appendix*, Fig. S2*A*). Also, recombinant PTHrP (10 ng/mL) affected neither thermogenic gene expression in 3T3-L1 adipocytes and iBACs (Fig. 2 *A* and *B*) nor *Pgc-1α* mRNA levels in primary white adipocytes isolated from iWAT or gWAT (*Ucp-1* cycle threshold >35) (*SI Appendix*, Fig. S2*B*). Since PTHrP utilizes a G_s_ protein–coupled receptor pathway to activate adenylate cyclase and induce adipocyte browning and lipolysis via cAMP (4, 28), we also measured glycerol release from 3T3-L1 adipocytes. At PTHrP concentrations up to 80-fold higher than observed in the plasma of cachectic mice (Fig. 1 *A* and *B*) (4), we observed a slight but not significant induction of lipolysis in 3T3-L1 adipocytes (1.63-fold) and primary adipocytes isolated from iWAT (1.42-fold) or gWAT (1.27-fold) (Fig. 2*C* and *SI Appendix*, Fig. S2*C*) but no effect on lipolysis in iBACs (Fig. 2*D*). IL-6 applied at concentrations 200-fold higher than observed in plasma of cachectic mice (Fig. 1 *A* and *B*) (11) had no measurable impact on glycerol release from 3T3-L1 adipocytes or iBACs, while forskolin treatment robustly increased glycerol release in both adipocyte models (4.5-fold in 3T3-L1 and 3.6-fold in iBACs) (Fig. 2 *C* and *D*). Thus, at physiological or pathological concentrations as observed in CAC, PTHrP and IL-6 do not directly stimulate thermogenic gene expression in adipocytes.

### Enhanced Intraadipose Sympathetic Activity Is Responsible for Increased β-Adrenergic Activation and Adipose Tissue Browning in CAC.

**Fig. 3.**
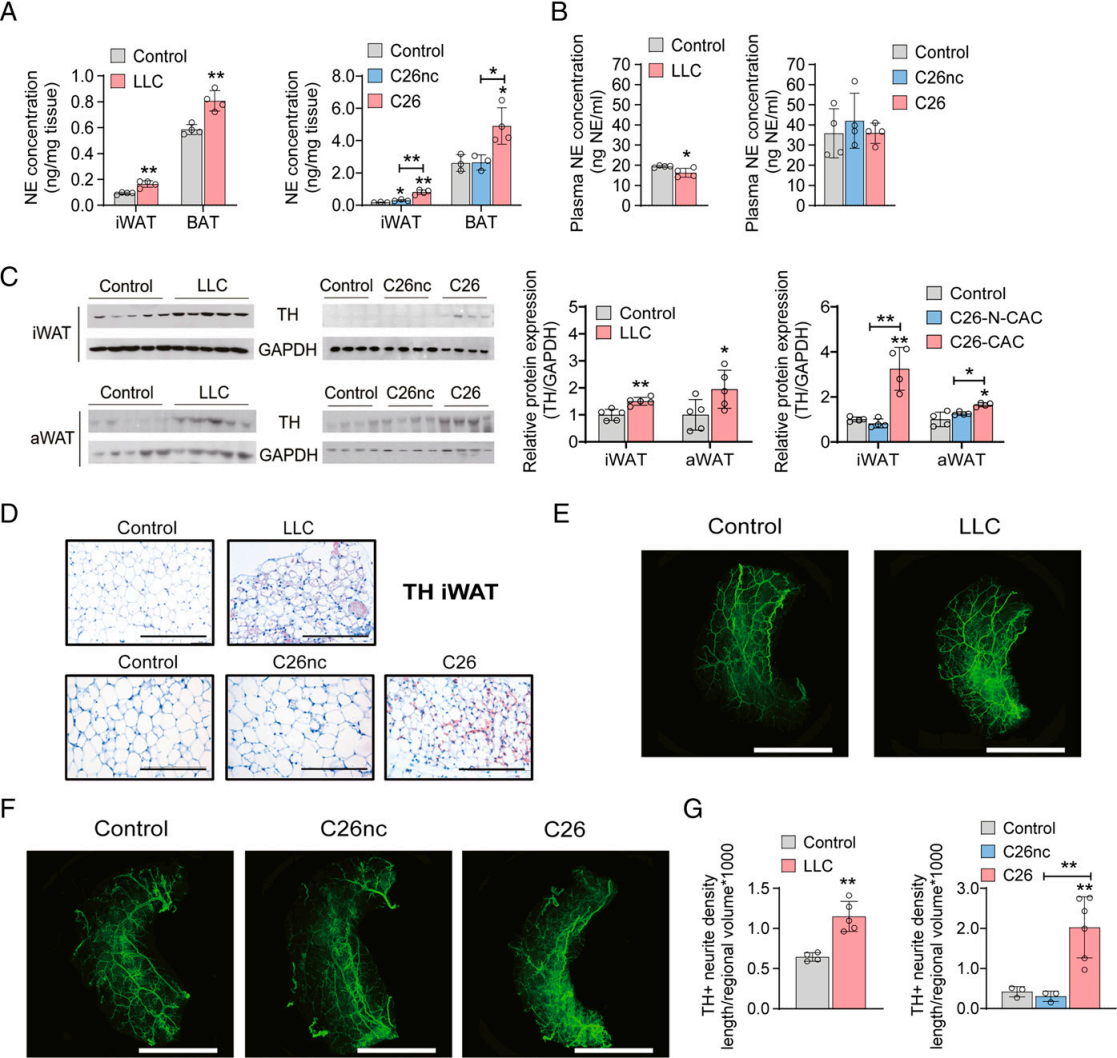
CAC is associated with increased catecholamine concentrations and elevated tyrosine hydroxylase (TH) expression in adipose tissue. (*A*–*D*) Mice bearing LLC, C26, or C26nc tumors were euthanized 14 to 16 d after tumor cell inoculation. (*A* and *B*) Noradrenaline (NE) contents in iWAT and BAT sections (*A*) as well as in plasma (*B*) of LLC, C26, and C26nc tumor-bearing mice were determined by ELISA (*n* = 3 to 4 per group). (*C*) Western blotting analysis to detect TH protein expression in iWAT and aWAT of LLC, C26, and C26nc tumor-bearing mice. Glycerinaldehyde-3-phosphat-dehydrogenase (GAPDH) was used as loading control. The protein contents of TH or GAPDH were quantified by densitometric analysis. (*D*) Representative iWAT sections of LLC, C26, and C26nc tumor-bearing mice immunolabeled for TH. (Scale bars: 200 µm.) (*E* and *F*) Representative three-dimensional projections of whole iWAT depots from (*E*) LLC, (*F*) C26, and C26nc tumor-bearing mice immunolabeled for TH and imaged at 1.26× magnification on a light-sheet microscope. (Scale bars: 5,000 µm.) (*G*) For quantification, randomly selected cubes were isolated from adipose tissue blocks that were derived from identical positions of whole adipose tissue depots of three to six mice per group. Neuron density in each cube was calculated as the ratio of total neurite length per regional volume. The mean of five cubes from each tissue block was calculated and represents one data point in the graph. Data are presented as means ± SD. Significance was determined by unpaired two-tailed Student’s *t* test or a one-way ANOVA followed by Tukey’s post hoc analysis. *P ≤ 0.05; **P ≤ 0.01.

The classical pathway of BAT activation and thermogenesis as well as WAT browning during chronic cold exposure involves the sympathetic stimulation of β3-adrenergic receptors by norepinephrine (NE) (29, 30). Our previous work, showing that β3-adrenoreceptor antagonism decreases WAT browning and ameliorates cachexia in the genetic keratin 5-son of sevenless (K5-SOS) model of CAC (3), argued for the involvement of β-adrenergic signaling in adipocytes during WAT browning causing the pathogenesis of CAC. Hence, we examined the concentration and cellular origin of catecholamines in WAT and BAT of mice suffering from CAC. Compared with control mice, NE concentrations were 2.3- and 1.4-fold higher in iWAT and BAT, respectively, of LLC tumor-bearing mice as well as 5.7- and 1.9-fold higher in iWAT and BAT, respectively, in C26 tumor-bearing mice (Fig. 3*A*). iWAT of C26nc tumor-bearing mice exhibited a 1.6-fold increase in NE concentrations compared with iWAT of control mice (Fig. 3*A*). Plasma concentrations of NE were not increased in cachectic mice, suggesting a local accrual of catecholamines in adipose tissue (Fig. 3*B*). Increased local catecholamine concentrations accompanied a robust increase in protein abundance of the rate-limiting enzyme for catecholamine biosynthesis, tyrosine hydroxylase (TH), in iWAT and aWAT of cachectic LLC (1.5-fold in iWAT and 1.8-fold in aWAT) and C26 tumor-bearing mice (3-fold in iWAT and 1.5-fold in aWAT) compared with control or C26nc tumor-bearing mice (Fig. 3*C*). Immunohistochemistry (IHC) confirmed the results of Western blotting analyses showing increased TH abundance in iWAT of cachectic LLC and C26 but not of C26nc tumor-bearing mice (Fig. 3*D*). While expression of TH was drastically increased, expression of the rate-limiting enzyme for catecholamine degradation, monoamine oxidase A, was unchanged in iWAT and aWAT (*SI Appendix*, Fig. S3*A*), indicating that the elevated catecholamine content in cachectic WAT is rather caused by increased synthesis than decreased degradation.

TH activity in WAT has been attributed to enzyme expression in adipocytes, sympathetic nerves, and alternatively, activated macrophages (31–33). To identify the cell type of TH expression and NE synthesis, we performed Western blotting analyses of cell fractions isolated from iWAT and BAT. TH was exclusively expressed in the stromal vascular fraction (SVF) but not in the adipocyte fraction of WAT and BAT (*SI Appendix*, Fig. S3*B*). Moreover, we were unable to detect TH mRNA (cycle threshold >35) or protein concentrations in primary peritoneal macrophages after treatment with 20 ng/mL interleukin-4 (IL-4). These results argued against adipocytes or activated macrophages as the primary source of catecholamines and prompted us to analyze WAT innervation.

Whole-mount immunolabeling and fluorescence microscopical imaging exposed a network of TH-positive fibers in white and brown adipose depots of control animals, which was markedly increased in iWAT (1.8- and 4.8-fold) (Fig. 3 *E*–*G*; Movies S1–S5; and *SI Appendix*, Fig. S3*C*) and BAT (*SI Appendix*, Fig. S3*D*) of LLC and C26 but not C26nc tumor-bearing mice, respectively. These results strongly suggested that elevated TH protein and catecholamine content in adipose tissue of cachectic mice originate from increased sympathetic activity due to increased TH expression in preexisting neurons and/or enhanced neurite outgrowth and are reminiscent of similar results from studies in cold exposed mice (34, 35). To find out whether peripheral catecholamine production is essential for WAT browning in CAC, we generated tamoxifen-inducible peripheral dopamine β-hydroxylase–deficient mice (DBHΔper mice) to conditionally block catecholamine synthesis in peripheral tissues but not in the brain (*SI Appendix*, Fig. S4*A*). Tamoxifen-treated DBHΔper mice had reduced dopamine β-hydroxylase (DBH) protein levels in iWAT and BAT but normal levels in the brain, and they exhibited similar food intake, body temperature, and WAT depot mass as control mice under ambient housing conditions (*SI Appendix*, Fig. S4 *B*–*E*). As a functional control experiment, we challenged DBHΔper mice with a 7-d cold exposure to activate β-adrenergic signaling. Cold-exposed DBHΔper mice exhibited reduced hormone-sensitive lipase (HSL) phosphorylation, diminished UCP-1 expression (*SI Appendix*, Fig. S4*F*), decreased body temperature (*SI Appendix*, Fig. S4*D*), and ameliorated adipose tissue loss (*SI Appendix*, Fig. S4*E*), indicating that defective β-adrenergic signaling led to decreased lipolysis and thermogenesis in these animals.

**Fig. 4.**
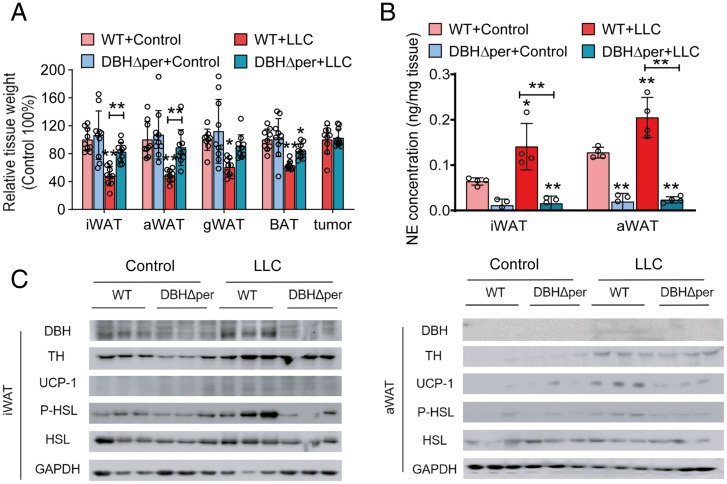
Deletion of dopamine β-hydroxylase (DBH) in peripheral tissues results in catecholamine depletion, reduced lipolysis, and impaired browning in adipose tissue of cachexigenic tumor-bearing mice. (*A*–*C*) LLC tumor-bearing mice were euthanized 14 to 16 d after tumor cell inoculation. (*A*) Tissue weights of DBHΔper LLC tumor-bearing mice relative to WT control animals (*n* = 9 to 10 per group; results of two independent experiments were combined). (*B*) NE contents of iWAT and aWAT of WT and DBHΔper LLC tumor-bearing mice and the respective control mice were determined by ELISA (*n* = 4 per group). (*C*) Western blotting analysis to detect DBH, tyrosine hydroxylase (TH), uncoupling protein-1 (UCP-1), phosphorylated (P)-HSL (Ser660), and HSL protein contents in iWAT and aWAT of LLC tumor-bearing and control WT and DBHΔper mice. Glycerinaldehyde-3-phosphate-dehydrogenase (GAPDH) was used as the loading control. Data are presented as means ± SD. Significance was determined by two-way ANOVA followed by Tukey’s post hoc analysis. *P ≤ 0.05; **P ≤ 0.01.

When DBHΔper mice were challenged with LLC, peripheral DBH deficiency also ameliorated CAC. Injection of LLC cells into wild-type (WT) and DBHΔper mice resulted in similar tumor burden, but only WT mice lost large amounts of adipose tissue (−52.3% iWAT, −51.5% aWAT, −39.6% gWAT, −37.0% BAT) (Fig. 4*A*). DBHΔper mice maintained normal iWAT, aWAT, and gWAT mass and only lost a minor amount of BAT mass (−16.8%) (Fig. 4*A*). In accordance with defective catecholamine synthesis, NE concentrations in iWAT and aWAT were 66% and 85% lower in DBHΔper than in WT mice, respectively, and did not increase in LLC tumor-bearing mice (Fig. 4*B*). Reduced β-adrenergic activation prevented phosphorylation and activation of HSL and the up-regulation of UCP-1 protein levels in iWAT and aWAT depots of LLC-bearing DBHΔper mice compared with LLC tumor-bearing WT mice (Fig. 4*C*). These results indicate that, similar to cold exposure, catecholamine production by peripheral sympathetic nerves drives lipid catabolism, WAT browning, and adipose tissue atrophy in CAC.

### Type 2 Immunity Contributes to Adipose Tissue Browning by Regulating Sympathetic Neurite Outgrowth in CAC.

**Fig. 5.**
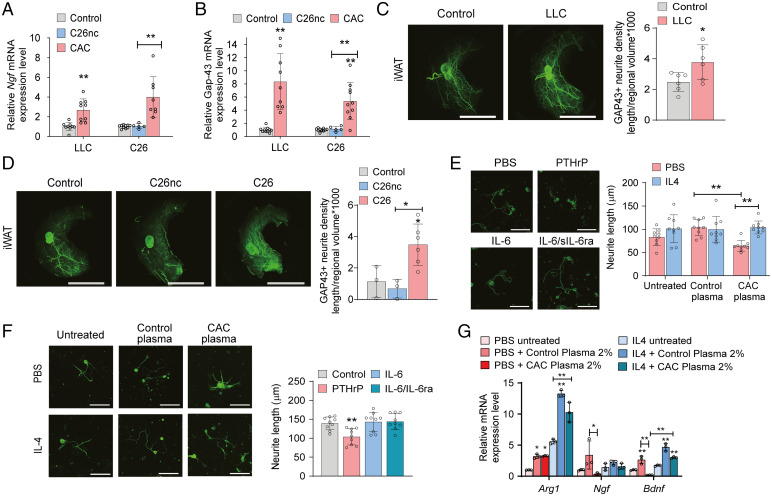
Alternatively activated macrophages promote sympathetic neurite outgrowth in CAC. (*A*–*D*) Mice bearing LLC, C26, or C26nc tumors were euthanized 14 to 16 d after tumor cell inoculation. (*A* and *B*) qRT-PCR analysis was used to detect mRNA levels of *Ngf* (*A*) and *Gap-43* (*B*) in iWAT (*n* = 5 to 10 per group; results from two independent experiments were combined). (*C* and *D*) Representative three-dimensional projections of iWAT depots from LLC (*C*), C26, and C26nc (*D*) tumor-bearing mice immunolabeled for GAP-43 and imaged at 1.26× magnification using a light-sheet microscope. For quantification, randomly selected cubes were isolated from adipose tissue blocks that were derived from identical positions of whole adipose tissue depots of three to six mice per group. Neuron density in each cube was calculated as the ratio of total neurite length per regional volume. The mean of five cubes from each tissue block was calculated and represents one data point in the graph. (Scale bars: 5,000 µm.) (*E* and *F*) Representative images of cultured primary sympathetic neurons grown in the presence or absence of 10 ng/mL PTHrP, 20 ng/mL IL-6, or 20 ng/mL IL-6/37.14 ng/mL (equimolar to IL-6) sIL-6ra (*E*) or cocultured with primary macrophages in the presence and absence of IL-4 and 2% plasma from control or LLC tumor-bearing (CAC) mice (*F*), immunolabeled for TH, and imaged by confocal fluorescence microscopy. Neurite length was determined using the Neuron J plug-in of ImageJ (*n* = 9 per condition). (Scale bars: 100 µm.) (*G*) Primary macrophages were incubated in the presence and absence of IL-4 and 2% plasma from control or LLC tumor-bearing (CAC) mice for 24 h and analyzed for neurotrophic gene expression using qRT-PCR (*n* = 3 per condition). Data are presented as means ± SD. Significance was determined by unpaired two-tailed Student’s *t* test or a one-way ANOVA (*A* and *E*) or by a two-way ANOVA followed by Tukey’s post hoc analysis (*F* and *G*). *P ≤ 0.05; **P ≤ 0.01.

Next, we focused on the mechanisms underlying increased sympathetic activity in WAT of cachectic animals. To assess whether neurite outgrowth contributed to the neuronal activity observed in whole-mount immunofluorescence imaging, we determined the expression of neurotrophic factors and their target genes in iWAT of cachectic and noncachectic mice. mRNA expression level of neurotrophic growth factor (*Ngf*) was significantly higher in iWAT of LLC (2.7-fold) and C26 (4.0-fold) tumor-bearing mice than in controls or C26nc tumor-bearing mice (Fig. 5*A*). The mRNA concentration of another key neurotrophin, brain-derived neurotrophic factor (*Bdnf*), was below the detection limit (cycle threshold >35 in real-time PCR) in iWAT tissue samples. Growth-associated protein-43 (GAP-43) is the primary downstream target of neurotrophins and an important mediator of neurite growth (36). mRNA expression of *Gap-43* was 8.3- and 5.4-fold higher in iWAT of LLC and C26 tumor-bearing mice compared with control animals and C26nc tumor-bearing mice, respectively (Fig. 5*B*). Increased transcription also translated into 1.5- and 3.6-fold higher GAP-43 protein abundance in whole-mount immunolabeling of iWAT of LLC (Fig. 5*C* and *SI Appendix*, Fig. S5*A*) and C26 (Fig. 5*D* and *SI Appendix*, Fig. S5*A*) tumor-bearing cachectic compared with control and noncachectic tumor-bearing mice, respectively, indicating increased neurotrophic signaling and neurite outgrowth in iWAT of cachectic mice.

To explore whether circulating factors directly promote neurotrophic signaling in CAC, we treated primary sympathetic neurons with plasma from control and cachectic mice and monitored neurite outgrowth. Primary sympathetic neurons that were treated with plasma from control mice arborized similar to untreated neurons, but addition of plasma from cachectic mice reduced neurite outgrowth by 24.8% (*SI Appendix*, Fig. S5*B*). Adding recombinant IL-6 (20 ng/mL) or IL-6 in combination with sIL-6ra (37.14 ng/mL, equimolar to IL-6) did not change neurite outgrowth, and recombinant PTHrP (10 ng/mL) significantly reduced arborizations of primary sympathetic neurons (−25.6%) (Fig. 5*E*). These results excluded the possibility that increased innervation of cachectic adipose tissue is mediated by a direct effect of IL-6 or PTHrP. Instead, they suggested that other adipose tissue–resident cells, like macrophages, indirectly promote neurite outgrowth.

Macrophages are the most abundant immune cell population in adipose tissue, and they have been linked to neuron function by releasing neurotrophins in many different tissues (37, 38). Performing IHC analyses of iWAT using an F4/80 antibody, we encountered a sharply increased number of macrophages in cachectic LLC (25-fold) and C26 (15-fold) tumor-bearing mice compared with control animals and C26nc tumor-bearing mice, confirming previous observations (*SI Appendix*, Fig. S5*C*) (3, 39). qRT-PCR analysis of iWAT revealed elevated mRNA expression of the type 2 immune marker genes arginase-1 (*Arg1*; 4.4-fold), interleukin-10 (*Il-10*; 3.8-fold), and transforming growth factor-β1 to -3 (*Tgfß1* to -*3*; 4.6-, 2.4-, and 2.4-fold, respectively) in LLC tumor-bearing animals compared with controls. No difference was observed in the mRNA expression level of the inflammatory type 1 marker gene *iNos* (*SI Appendix*, Fig. S5*D*). Similarly, iWAT of C26 tumor-bearing animals exhibited higher expression of *Arg1* (6.6-fold), *Il-10* (2.4-fold), and *Tgfß3* (1.8-fold) but not of *Tgfß1* and *-2* and *iNos* mRNA levels compared with control mice (*SI Appendix*, Fig. S5*D*).

To investigate whether a direct cross-talk between macrophages and neurons regulates neurite outgrowth, we established transwell-based cell coculture experiments using primary macrophages and primary sympathetic neurons. The effect of alternatively activated macrophages on neuronal outgrowth was addressed by preincubating primary macrophages without plasma (untreated), with 2% plasma derived from normal control mice (control plasma), or with 2% plasma derived from cachectic LLC tumor-bearing mice (CAC-plasma) in the respective presence and absence of recombinant IL-4. After preincubation, the transwell inserts containing macrophages were transferred to primary neuron cultures, and neurons were analyzed for TH-positive arborizations by immunofluorescence microscopy. Neurons cocultured with macrophages that were preincubated with CAC-plasma in the absence of IL-4 exhibited 38.8% reduced arborizations compared with neurons cocultured with macrophages pretreated with control plasma (Fig. 5*F*), again arguing for the presence of an inhibitory factor on neuron growth in the plasma of cachectic mice. In contrast, coculture of neurons with macrophages that were pretreated with CAC-plasma in the presence of IL-4 increased neurite outgrowth, overriding the inhibitory effect of CAC-plasma on neurite growth (Fig. 5*F*). The presence of IL-4 significantly increased mRNA levels of the neuroprotective factor *Arg1* (37, 40) in untreated, control plasma–treated, and CAC-plasma–treated macrophages by 5.6-, 4.1-, and 3.2-fold, respectively (Fig. 5*G*). Similarly, compared with untreated or control plasma-treated macrophages, mRNA levels of the major neurotrophins *Ngf* and *Bdnf* were decreased in CAC-plasma–treated macrophages in the absence of IL-4 (−77.8 and −81.5%, respectively) but were recovered upon IL-4 treatment to higher levels than in untreated macrophages (Fig. 5*G*). These data strongly support the concept that alternatively activated macrophages express neurotrophic factors to generate a neuroprotective environment that promotes WAT innervation in cachectic mice.

**Fig. 6.**
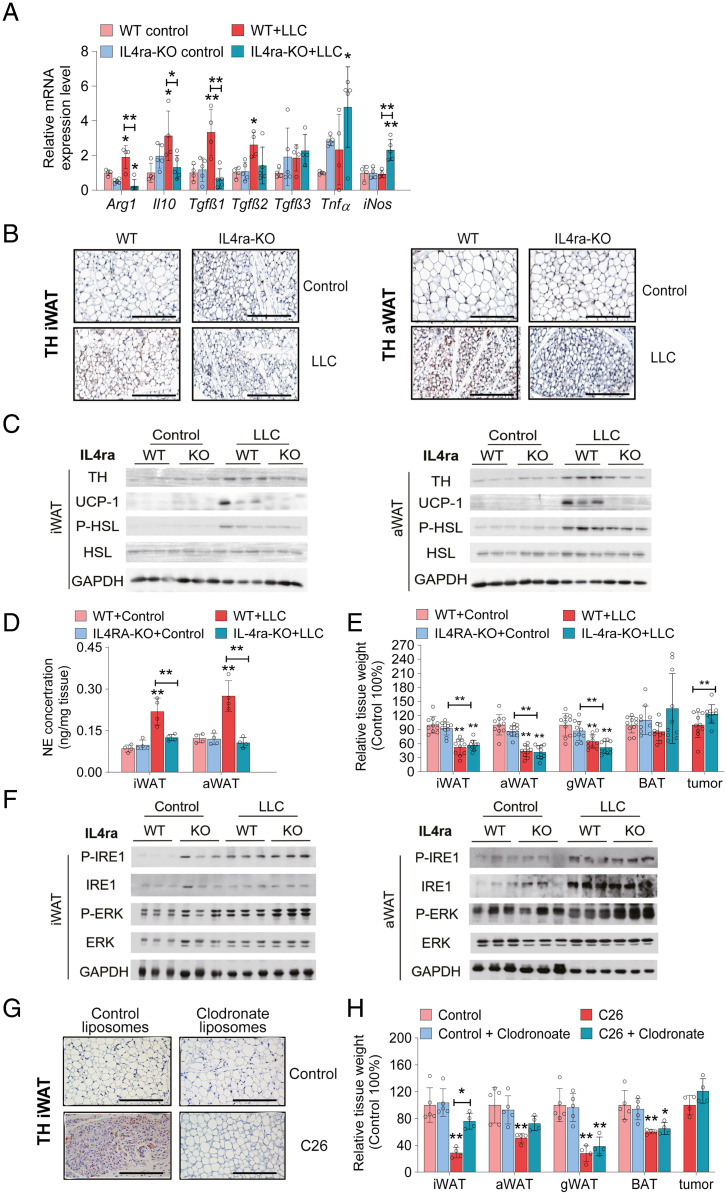
IL-4ra deficiency or local macrophage depletion prevents CAC-induced catecholamine synthesis and browning of adipose tissue. (*A*–*F*) WT and IL4ra-knock out (KO) mice bearing LLC tumors were euthanized 14 to 16 d after tumor cell inoculation. (*A*) qRT-PCR analysis to detect mRNA levels of type 1 and type 2 immune cell marker genes in iWAT (*n* = 4 to 5 per group). (*B*) Representative iWAT and aWAT sections immunolabeled for tyrosine hydroxylase (TH). (Scale bars: 200 µm.) (*C*) Western blotting analysis to detect TH, uncoupling protein-1 (UCP-1), phosphorylated (P) (Ser660) hormone sensitive lipase (P-HSL), and HSL protein contents in iWAT and aWAT. Glycerinaldehyde-3-phosphat-dehydrogenase (GAPDH) was used as loading control. (*D*) Norepinephrine (NE) contents in iWAT and aWAT were determined by ELISA (*n* = 4 per group). (*E*) Tissue weights of WT and IL4ra-KO LLC tumor-bearing mice relative to tissue weights of control animals (*n* = 10 per group; results of two independent experiments were combined). (*F*) Western blotting analysis to detect phosphorylated (P) (Ser724)-inositol-requiring protein-1 (P-IRE1), IRE1, phosphorylated (P) (Thr202/Tyr204)-extracellular signal–regulated kinase (P-ERK), and ERK protein expression in iWAT and aWAT depots. (*G* and *H*) C26 tumor-bearing mice and control mice were subcutaneously injected with control/clodronate liposomes every 2 d and euthanized 14 to 16 d after tumor cell inoculation. (*G*) Representative iWAT sections of control and C26 tumor-bearing mice treated with clodronate/control liposomes immunolabeled for TH. (Scale bars: 200 µm.) (*H*) Tissue weights of control/clodronate liposomes–treated C26 tumor-bearing mice relative to tissue weights of the respective control animals (*n* = 4 to 5 per group). Data are presented as means ± SD. Significance was determined by two-way ANOVA followed by Tukey’s post hoc analysis. *P ≤ 0.05; **P ≤ 0.01.

To verify the crucial role of IL-4–mediated alternative activation of macrophages for sympathetic activity of adipose tissue during CAC in vivo, we pursued two strategies. First, we studied interleukin-4 receptor-α–deficient mice (IL-4ra-KO) in comparison with WT littermates in terms of adipose innervation, WAT browning, and adipose tissue loss in the LLC cachexia model. In line with diminished IL-4 signaling, IL-4ra-KO mice exhibited low expression levels of the alternative macrophage marker genes *Arg1* and *Il-10*, which furthermore, were not enhanced in iWAT in the presence of the cachexigenic LLC tumor (Fig. 6*A*). In IHC and Western blotting analyses, we observed reduced TH protein abundance (Fig. 6 *B* and *C*) and decreased NE concentrations (Fig. 6*D*) in iWAT (−48%) and aWAT (−56%) of LLC tumor-bearing IL-4ra-KO animals compared with WT controls. Consistent with a decreased β-adrenergic tone, UCP-1 protein content was reduced in iWAT and aWAT depots of LLC tumor-bearing IL-4ra-KO mice compared with WT depots (Fig. 6*C*), corroborating the conclusion that IL-4ra–dependent IL-4 activation of macrophages regulates sympathetic activity and WAT browning. However and in contrast to peripheral DBH deficiency, IL-4ra deficiency did not reduce HSL phosphorylation or adipose tissue atrophy of tumor-bearing mice, despite reduced β-adrenergic signaling (Fig. 6 *C* and *E*). These results also differ from the experimental outcome of cold exposure experiments with IL-4ra-KO mice. There, we saw increased TH, UCP-1, and phosphorylated HSL protein contents in iWAT and aWAT of cold-exposed WT but not IL-4ra-KO mice (*SI Appendix*, Fig. S6*A*). Reduced lipolysis and thermogenesis ameliorated cold-induced adipose tissue loss by 21.1% in iWAT and 12.2% in aWAT, respectively, in IL-4ra-KO animals (*SI Appendix*, Fig. S6*B*).

Searching for lipolysis activation pathways that are independent of the β-adrenergic–cAMP–protein kinase A (PKA) signaling cascade, we found increased *TNFα* and *iNos* mRNA expression levels in iWAT of LLC-bearing IL-4-ra-KO animals compared with tumor-bearing WT mice (Fig. 6*A*). TNFα-mediated inflammatory signaling has been shown to stimulate lipolysis via increasing endoplasmic reticulum (ER) stress component inositol-requiring protein-1 (IRE1) kinase activity, leading to extracellular signal–regulated kinase (ERK) activation and HSL phosphorylation (41, 42). In accordance with this signaling pathway, we observed stronger IRE1 and ERK phosphorylation in iWAT and aWAT depots of LLC tumor-bearing IL-4ra-KO mice than in iWAT and aWAT of WT mice (Fig. 6*F*). Hence, IL-4ra deficiency, by impairing type 2 immune cell activation, reduces β-adrenergic signaling and browning, but IRE1/ERK-dependent activation of lipolysis prevents the protective effect of a reduced β-adrenergic tone on adipose atrophy in tumor-bearing IL-4ra–deficient animals.

In a second approach, we employed clodronate injection experiments to locally and selectively deplete subcutaneous adipose tissue macrophages. Clodronate liposomes or control liposomes (vehicle) were subcutaneously injected into iWAT depots of C26 tumor-bearing mice. Clodronate treatment largely diminished F4/80 expression in control and cachectic iWAT, demonstrating local macrophage depletion (*SI Appendix*, Fig. S6*C*). Importantly, TH, UCP-1, and phosphorylated HSL protein abundance were sharply decreased in iWAT upon macrophage depletion (Fig. 6*G* and *SI Appendix*, Fig. S6*D*). Consequently, iWAT tissue weight was increased 2.6-fold in clodronate-treated C26 tumor-bearing mice compared with vehicle-treated cachectic mice (Fig. 6*H*). The protective effects of clodronate were less pronounced in aWAT of C26 tumor-bearing mice, indicating a locally limited effect of the drug only at the site of injection (*SI Appendix*, Fig. S6*D*).

Overall, our results demonstrate that adipose tissue remodeling during CAC is dependent on type 2 immune cells generating a neuroprotective environment. The increased sympathetic activity promotes β-adrenergic activation and browning of adipose tissue in CAC.

## Discussion

Cachexia is a life-threatening condition that occurs at the end stage of various unrelated diseases, such as infectious diseases, autoimmune diseases, heart failure, and cancer (2). The molecular triggers that initiate cachexia as well as the mechanisms that lead to hypermetabolism, depletion of energy stores and adipose tissue, and muscle wasting remain incompletely understood. Inflammation plays an important role in the pathogenesis of many types of cachexia, including CAC, where mixtures of inflammatory cytokines, including TNFα and members of the IL-6 family (IL-6, LIF, and ciliary neurotrophic factor) and the TGF-β family (GDF-8, -11, and -15), as well as PTHrP are considered to elicit the wasting disorder (43).

In adipose tissue, different murine models of CAC including C26 and LLC tumors provoke an IL-6– and/or PTHrP-dependent catabolic state characterized by massive lipolytic fat degradation and BAT activation as well as WAT browning, all contributing to increased resting energy expenditure (3, 4). In the present study, we show that a complex immune-sympathetic neuron cross-talk promotes the metabolic switch in WAT, leading to adipose tissue wasting and CAC. Our conclusion is based on the findings that IL-6 and PTHrP are essentially unable to directly activate thermogenic gene expression in adipocytes. Instead, they activate immune cells, which leads to increased sympathetic activity, catecholamine synthesis, and browning in C26 (with high IL-6/PTHrP plasma levels) but not in C26nc (with low IL-6/PTHrP plasma levels) tumor-bearing mice. Our observation that IL-6 and PTHrP act indirectly by activating immune cells contradicts previous studies claiming that cachexokines initiate the “catabolic” state in adipocytes directly (4, 44). A conceivable explanation for the divergent findings may reside in the fact that SVF-derived adipocyte cultures used in previous studies contain nonadipose cell types (macrophages, fibroblasts, and others) that may contribute to the indirect effect of cachexokines (45, 46).

Sympathetic neuron-derived NE is a classical activator of lipolysis, BAT activity, and WAT browning in response to cold exposure (47). We now demonstrate that high neuronal TH expression and elevated catecholamine synthesis in WAT trigger the catabolic state in CAC. This conclusion is consistent with previous observations suggesting that β-adrenergic signaling contributes to the pathogenesis of CAC in mice and humans (3). For instance, we previously showed that denervation of BAT reduces UCP-1 expression and weight loss in both healthy and cachectic mice. Consistent with this observation, β3-adrenergic antagonism ameliorated body weight, fat, and muscle loss as well as browning in a genetic mouse model of CAC (3). Furthermore, clinical trials demonstrated that β-blocker administration prevents loss of fat mass, attenuates WAT remodeling, and protects from WAT and body weight loss in advanced CAC (48), severe burn trauma (23), and chronic heart failure (49), respectively. Our results demonstrate that increased sympathetic activity based on increased expression of TH in preexisting neurons and/or increased sympathetic innervation determines the hypermetabolism in WAT, providing a plausible mechanistic basis for WAT atrophy not only in CAC, but also in cachexia associated with burn trauma or chronic heart disease. Consistent with this concept, absence of triggers, such as IL-6, or blockade of peripheral catecholamine synthesis in DBH-deficient mice prevents β-adrenergic activation and ameliorates CAC (3).

Sympathetic neurons represent the predominant cell type expressing TH and synthesizing catecholamines (32). However, TH activity and catecholamine production have also been reported in other cell types of WAT, including adipocytes and alternatively activated macrophages (31, 33). Adipose tissue–resident, alternatively activated macrophages have also been shown to contribute to WAT browning of cold-exposed, burn-injured, or calorie-restricted mice (33, 50, 51). We were unable to detect TH expression or activity in either adipocytes or macrophages, and therefore, in accordance with the work of Fischer et al. (34), we consider sympathetic neurons to be the major source of NE in our mouse models of CAC. Although macrophages are not directly involved in catecholamine synthesis, our study suggests that IL-6–mediated alternative activation of macrophages (52, 53) promotes innervation and metabolic reprogramming of WAT during CAC.

Macrophage-sympathetic neuron coculture experiments and the characterization of CAC in IL-4ra-KO mice indicated that in inflammatory conditions like CAC, noninflammatory, IL-4–activated macrophages are recruited to render a supportive microenvironment for axon regeneration. To promote neuron growth, type 2 macrophages produce neurotrophins and increase ARG1-mediated polyamine synthesis and secretion (54). Depletion of WAT-resident macrophages by clodronate injection (55) or blocking type 2 immune cell activation by IL-4ra deletion impedes the establishment of a neuroprotective environment to preserve neurite outgrowth of sympathetic nerves. Similar mechanisms have recently been observed for inflammation-induced neuronal damage in the gastrointestinal tract or spinal cord injury, where alternatively activated neuroprotective macrophages enhance neuron arborization (37, 38, 56). Additionally, increased TH protein expression in already preexisting neurons may also contribute to the apparent neuronal density in volume fluorescence imaging analyses (57). Together, this increased neuronal activity leads to enhanced local catecholamine synthesis, β-adrenergic stimulation of WAT, and WAT browning.

Notably, while IL-4ra deficiency protects WAT from excessive innervation and browning in the LLC cachexia model, it does not protect from increased lipolysis or adipose tissue atrophy. This suggested that in addition to β-adrenergic stimulation, other mechanisms contribute to increased triglyceride catabolism in this mouse model, and we found that, indeed, inflammation-induced ER stress likely driven by TNFα underlies elevated lipolysis in WAT of IL-4ra-KO mice. However, other to be identified signaling pathways may additionally contribute to the regulation of lipid degradation and adipose atrophy in IL-4ra–deficient mice.

**Fig. 7.**
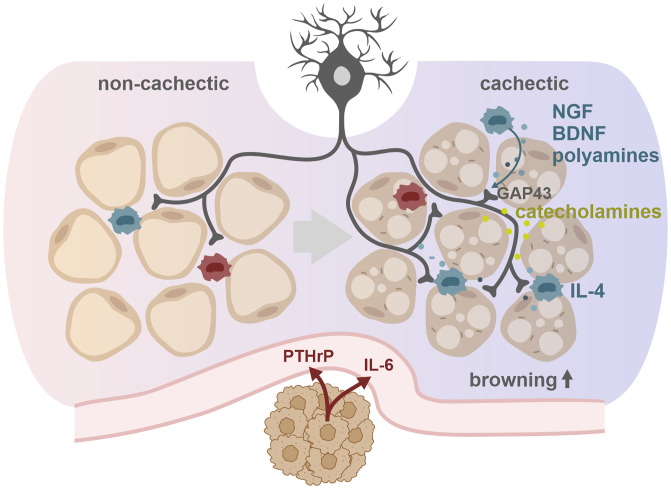
Mechanisms of WAT browning in CAC. A chronic inflammatory, hypercatabolic state causes metabolic adaptations in WAT, summarized in the term browning, which contribute to WAT atrophy. We demonstrate that cachexia-associated proinflammatory factors, like IL-6 and PTHrP, do not directly induce a catabolic switch in adipocytes but likely activate an intraadipose macrophage-sympathetic neuron cross-talk that orchestrates browning of WAT. The proinflammatory environment, generated by the cachexigenic tumor, causes the infiltration and IL-4–dependent alternative activation of macrophages (blue) in WAT, whereas the amount of proinflammatory macrophages (red) was unaffected. Alternatively activated macrophages secrete NGF, BDNF, and polyamines to generate a neuroprotective environment that allows for an increased GAP43-conducted outgrowth and activity of sympathetic neuronal projections. Enhanced sympathetic activity results in increased neuronal catecholamine secretion, β-adrenergic activation of adipocytes, and WAT browning.

Although our study highlights a key role of macrophage-sympathetic neuron cross-talk in the metabolic switch toward WAT catabolism in mice with CAC, a number of important questions remain open. These include, for example, whether PTHrP, similar to IL-6, indirectly increases WAT browning by targeting immune cells or whether the described mechanisms are also relevant for other IL-6/PTHrP–independent types of CAC. Also, the current study focused on the effects of β-adrenergic signaling and macrophages in adipose tissue loss during CAC. Additional, for example, genetic models of CAC with more severe muscle loss than observed in the xenotransplant models of LLC or C26 should be investigated to elucidate a potential role of the neuron–macrophage cross-talk in muscle biology during CAC. Another important question concerns mechanisms of noncancer types of cachexia as observed in infectious, autoimmune, or heart diseases. The fact that β-adrenergic antagonism partially improved cachexia in patients affected with some of these diseases (23, 24, 49) suggests a more general relevance of the mechanism described in our study. However, this notion has to be addressed experimentally. Taken together, we show that sympathetic innervation supported by a macrophage-mediated neuroprotective environment is essential for WAT browning and adipose atrophy in murine CAC (Fig. 7). Targeting this intraadipose communication axis between macrophages and sympathetic neurons may provide a therapeutic option to treat CAC, avoiding classical β-adrenergic receptor blockage and associated adverse cardiovascular side effects.

## Materials and Methods

### Cell Lines and Chemicals.

All growth media were supplemented with 10% fetal bovine serum (Thermo Fisher Scientific), 100 IU/L penicillin, and 0.1 mg/L streptomycin. LLC cancer cells (ATCC CRL-1642), C26 noncachexigenic cancer cells (Cell Resource Center for Biomedical Research, Tohuku University), and C26 (AMGEN) cancer cells were cultivated in Roswell Park Memorial Institute (RPMI) medium 1640 (A10491-01; Thermo Fisher Scientific).

The 3T3-L1 (ATCC CL-173) mouse embryonic fibroblasts were maintained in Dulbecco's Modified Eagle's Medium (DMEM) (2124931; Thermo Fisher Scientific). Cells were cultured at 37 °C in a 5% CO_2_ humidified atmosphere. Two days after confluency, 3T3-L1 cells were differentiated using 10 μg/mL insulin (I2643; Sigma-Aldrich), 0.25 μM dexamethasone (D4902; Sigma-Aldrich), and 0.5 mM isobutylmethylxanthine (I5879; Sigma-Aldrich). After 3 and 5 d, medium was supplemented with 10 and 0.05 μg/mL insulin, respectively.

SV40 iBACs (provided by J. G. Bogner-Strauss, Graz University of Technology, Graz, Austria) were cultured in DMEM also containing 20 mM 4-(2-hydroxyethyl)-1-piperazineethanesulfonic acid (HEPES). Confluent cells were differentiated using 0.5 mM 3-isobutyl-l-methylxanthine, 0.5 μM dexamethasone, 20 nM insulin, 1 nM triiodothyronine (T6397; Sigma-Aldrich), and 125 μM indomethacin (I8280; Sigma-Aldrich). Two days after induction, medium was changed to medium containing 20 nM insulin and 1 nM triiodothyronine.

### Ethical Approval.

All animal studies were approved by and performed according to the guidelines of the ethics committee of the University of Graz, the Austrian Federal Ministry for Science and Research and are in accordance with the Council of Europe Convention (GZ 66.007/0005-V/3b/2019).

### Animals.

Mice were kept on a regular light/dark cycle (14-h light/10-h dark) in a specific pathogen-free environment and fed a standard chow diet (M-Z extrudate, V1126; Ssniff). Inducible peripheral DBH-knockout mice were generated as described in *SI Appendix*. Tamoxifen-inducible Rosa26CreERT2 (MGI:3764519) mice were a gift from Anton Berns, Amsterdam, the Netherlands. IL-4ra-KO mice (MGI:2657172, C57Bl6J background) were a gift from Martin Gericke, Institute of Anatomy, Leipzig University, Leipzig, Germany. Ten- to 12-wk-old male C57BL/6J mice were anesthetized with isoflurane and injected with 0.5 × 10^6^ LLC cancer cells per 100 μL phosphate buffered saline (PBS) subcutaneously into the right flank; 0.5 × 10^6^ C26 or C26nc tumor cells per 100 μL PBS were subcutaneously injected into the right flank of isoflurane-anesthetized 10- to 12-wk-old male CD2F/1 mice. Control mice were injected subcutaneously with 100 μL PBS into the right flank. After 14 to 16 d, mice were anesthetized with isoflurane to collect blood and euthanized by cervical dislocation. For chronic cold exposure, mice were transferred from room temperature (RT; 21 °C to 22 °C) to prechilled cages at 4 °C with bedding, a cotton nestlet, free access to standard chow diet and water, and a partially opened cage lid. Rectal temperatures were recorded every 60 min for the first 6 h of cold exposure using a rectal probe. Food intake was determined on single housed mice by manually weighing the food every day at 10 AM. After 7 d of cold exposure, mice were anesthetized to collect blood and were euthanized by cervical dislocation. Control/clodronate liposomes (CLD-8901; Macrophage Depletion Kit; Encapsula NanoSciences) were subcutaneously injected into the iWAT depot of CD2F/1 mice. For each animal, 25 μL liposome emulsions were injected into iWAT on each side every 2 d after tumor cell injection.

### Primary Cell Isolation

#### Adipocytes and SVF.

iWAT and gWAT were excised and minced using a scalpel. Minced samples were placed in DMEM containing collagenase type 2 (LS004174; Worthington) at a final concentration of 2 mg/mL. Samples were incubated in an orbital shaker at 37 °C for 0.5 to 1 h. After digestion was completed, samples were passed through a sterile 70-µm cell strainer (352350; BD Biosciences) and diluted with DMEM to a final volume of 20 mL. The cell suspension was centrifuged at 200 × *g* and RT for 5 min. The upper fraction contained primary adipocytes, which were washed with PBS three times and used for further analysis. The lower fraction was centrifuged at 1,000 × *g* at RT for 5 min. The cell pellets were resuspended in 5 mL of red blood cell lysis buffer (158902; Qiagen), incubated for 3 min and RT, and then, washed three times using PBS. Cell pellets were collected as the SVF and used for further experiments.

#### Macrophages.

The isolation and culture of peritoneal macrophages were performed according to standard protocols (56). Adult mice were euthanized by cervical dislocation. Peritoneal macrophages were harvested by intraperitoneal lavage using 10 mL of ice-cold PBS. The lavage fluid was centrifuged at 500 × *g* and 4 °C for 10 min to pellet cells. Cell pellets were resuspended in 5 mL of red blood cell lysis buffer, incubated for 3 min at RT, and then, washed three times using PBS. After the final wash, cells were resuspended in RPMI 1640 full medium plated onto a 100-mm culture dish or six-well plate and maintained at 37 °C in a humidified incubator with 5% CO_2_.

#### Sympathetic neurons.

The isolation and culture of primary sympathetic neurons were performed according to standard protocols (58) and are described in detail in *SI Appendix*. In brief, murine superior cervical ganglions (SCGs) were excised and digested using collagenase and dispase. After centrifugation, washing, and filtration, dissociated neurons were plated on poly-d-lysine– and laminin-coated cover glass in wells of eight-well culture chambers containing L15 Leibovitz full media and B-27 plus supplement.

### Transwell Culture of Neurons and Macrophages.

For transwell-based neuron–macrophage cocultures, peritoneal macrophages were plated on a cell culture insert, placed in a 12-well plate, and maintained in 1 mL RPMI 1640 full medium (with 2% B-27 plus supplement). Macrophages were preincubated with 2% plasma derived from control or LLC tumor-bearing mice (passed through a 40-μm filter) in the presence and absence of recombinant IL-4 (20 ng/mL; I1020; Sigma-Aldrich). SCG neurons were plated on 0.01% poly-d-lysine– and 3 µg/mL laminin–coated cover glass in a 12-well plate and maintained in prewarmed L15 Leibovitz full media. SCG neurons were allowed to adhere to the coated cover glass for 2 h. Thereafter, the SCG neuron culture medium was replaced with macrophage culture medium, and the transwell inserts containing macrophages were transferred to the SCG neuron cultures. After 36 h, neuron cultures were fixed with 4% cold paraformaldehyde in PBS for 20 min. Subsequently, neurons were analyzed for TH-positive arborizations by immunofluorescence.

### Preparation of Conditioned Media.

For preparation of tumor cell conditioned media, cells were seeded in six-well plates and incubated in RPMI 1640 full medium. After cells approximately grew to 80% confluence, medium was changed to fresh medium. After 24 h, the medium was collected and centrifuged at 300 × *g* for 3 min at RT. The supernatant was collected and stored for further experiments. Plain medium was used as control.

### Glycerol Release of Cultured Adipocytes.

Primary white adipocytes were washed with PBS and incubated in Krebs–Henseleit buffer II (115 mM NaCl, 25 mM NaHCO_3_, 5.9 mM KCl, 1.18 mM MgCl_2_, 1.23 mM NaH_2_PO_4_, 6 mM glucose) containing 25 mM Hepes and 1% FA-free bovine serum albumin (BSA) (A6003; Sigma-Aldrich) in the presence or absence of 40 ng/mL PTHrP (4017147; Bachem) in an orbital shaker (50 rpm) at 37 °C for 2 h. After incubation, cells were placed on ice. An aliquot of the incubation medium was taken from underneath the cell layer, and glycerol content was measured using free glycerol reagent (F6428; Sigma-Aldrich) according to the manufacturer’s instructions. Fully differentiated 3T3-L1 adipocytes and iBACs were preincubated with 20 ng/mL IL-6 (I9646; Sigma-Aldrich), 37.14 ng/mL (equimolar to IL-6) sIL-6Rα (1830-SR; Bio-techne), or 40 ng/mL PTHrP for 8 h. Afterward, medium was changed to DMEM + 2% FA-free BSA (A6003; Sigma-Aldrich) and incubated for 2 h. As a positive control for stimulated lipolysis, cells were incubated in the presence of 20 μM forskolin (F6886; Sigma-Aldrich). Lipolysis was measured as the release of glycerol into the medium and determined using free glycerol reagent. After incubation, adipocytes were washed three times with PBS and lysed in 0.3 M NaOH/0.1% sodium dodecylsulfate (SDS) by shaking the plates for 3 h at RT. Protein concentration of cell lysates was determined using BCA reagent (23225; Thermo Fisher Scientific) and BSA as standard. Lipolysis of adipocytes was calculated as nanomoles of glycerol per milligrams of cell protein and hour.

### Quantification of PTHrP, IL-6, and NE Concentrations.

Plasma PTHrP (S-1227; Bachem), IL-6 (88-7064-22; Thermo Fisher Scientific), and NE (BA E-5200; Rocky Mountain Diagnostics) concentrations were quantified using enzyme-linked immunosorbent assay (ELISA), following the manufacturers’ protocol. Cell culture supernatants were centrifuged at 1,000 × *g* and 4 °C for 10 min, and the supernatant was collected for cytokine quantification. Adipose tissue was homogenized by sonication in homogenization buffer (0.01 N HCl, 1 mM ethylenediaminetetraacetic acid [EDTA], 4 mM sodium metabisulfite), and cellular debris was removed by centrifugation at 15,000 × *g* and 4 °C for 15 min. The homogenates were collected and stored at −80 °C.

### qRT-PCR Analysis.

Total RNA was extracted from snap-frozen tissues using TRIzol (15596; Thermo Fisher Scientific) reagent following the manufacturer’s instructions. The quality of RNA was verified by agarose gel electrophoresis. RNA concentrations were analyzed using a NanoDrop microvolume spectrophotometer (Thermo Fisher Scientific). To remove DNA contaminations, RNA was incubated with Dnase I (M0303S; New England Biolabs; 1 U/mL) at 25 °C for 15 min followed by heat inactivation of the enzyme at 65 °C for 10 min. Thereafter, 1 µg RNA was transcribed using random primers and a High-Capacity cDNA Reverse Transcription Kit (Applied Biosystems; Thermo Fisher Scientific). Gene expression analysis was performed by qRT-PCR using the StepOnePlus Real-Time PCR System (Thermo Fisher Scientific) and Universal SYBR green (172-5125; Thermo Fisher Scientific). The 2 to the power of negative delta-delta cycle threshold (2-ΔΔCT) method was used to quantify amplified fragments. Actin was used as an internal control for normalization. A list of primers used is shown in *SI Appendix*.

### Western Blotting Analysis.

Cell or tissue samples were lysed in solution A (0.25 M sucrose, 1 mM EDTA, 1 mM dithiotreitol [DTT]) containing 1 µg/mL pepstatin, 2 µg/mL antipain, 20 µg/mL leupeptin (Carl Roth GmbH & Co KG), and phosphatase inhibitors (PhosSTOP; Roche) by sonication (Sonoplus; Bandelin) or homogenization using an Ultra Turrax (IKA). Cell debris was removed by centrifugation at 1,000 × *g* and 4 °C for 10 min, and the fat-free infranatant was collected. Protein concentration was determined using the Bio-Rad protein assay; 20 µg protein was resolved by SDS-polyacrylamide gelelectrophoresis (PAGE). Thereafter, proteins were transferred onto a polyvinylidene fluoride transfer membrane (Carl Roth GmbH) in N-cyclohexyl-3-aminopropanesulfonic acid (CAPS) buffer (10 mM CAPS, 10% methanol, pH 11.0). After blocking the membrane with 10% blotting-grade milk powder (Carl Roth GmbH) in Tris-SodiumTween (TST) (50 mM tris(hydroxymethyl)aminomethan (Tris)⋅HCl, 0.15 M NaCl, 0.1% Tween-20, pH 7.4), the antibodies listed in *SI Appendix* were applied. Protein expression was visualized by enhanced chemiluminescence using Clarity Western ECL Substrate and the ChemiDoc Touch Imaging System (170-5061; Bio-Rad). Signal intensities were quantified by densitometric analyses using Image Lab software (version 5.2.1; Bio-Rad).

### IHC and Hematoxylin/Eosin Staining.

Formalin-fixed (4% and PBS-buffered), paraffin-embedded tissue samples were sliced and stained with hematoxylin and eosin according to standard histopathological techniques (59). For IHC analysis of adipose tissues, UCP-1 (ab10983, 1:100; Abcam), F4/80 (Serotec MCA 497 GA, 1:50; Bio-Rad), and TH (ab112, 1:200; Abcam) antibodies were used. Antibody binding was visualized using aminoethyl carbazole (3464; Dako).

### Immunofluorescence Staining.

Confocal laser scanning microscopy of cultured primary sympathetic neurons was performed on a Leica DMi8 CS DLS inverse research microscope equipped with an HC PL APO 20×/0.75 IMM CORR CS2 objective. TH was immunolabeled using a TH (AB152, 1:1,000; Millipore) primary antibody followed by an Alexa Fluor 647–conjugated secondary antibody (A-21246, 1:5,000; Thermo Fisher Scientific), which was excited at 633 nm and detected between 650 and 750 nm.

### Volume Fluorescence Imaging of Adipose Tissue.

Volume fluorescence imaging of adipose tissue depots was performed as previously reported (35) and is described in detail in *SI Appendix*. In brief, adipose tissue was dissected, fixed in 1% paraformaledhyde (PFA), dehydrated using methanol, and bleached with 5% H_2_O_2_. Thereafter, tissues were rehydrated using methanol and permeabilized in Triton X-100/dimethylsulfoxide (DMSO)/glycine. After blocking, tissues were stained with TH or GAP-43 antibodies and with Alexa dye–conjugated secondary antibodies. Immunolabeled iWAT was embedded in agarose blocks and dehydrated in methanol. Then, tissue blocks were incubated with dichloromethane and finally cleared using dibenzyl ether. Optically cleared iWAT was imaged on the LaVisionBiotec Ultramicroscope II. Image stacks were reconstructed using Imaris (https://imaris.oxinst.com/packages).

### Statistical Analysis.

Data are shown as means ± SDs. The number of mice is defined as “*n*.” Statistical analysis was performed on data distributed in a normal pattern between two groups by two-tailed Student’s *t* test and between more than two groups using one-way or two-way ANOVA followed by Tukey’s post hoc analysis using GraphPad Prism 6 (GraphPad Software). For all analyses, group differences were considered statistically different for *P* < 0.05 (*) or *P* < 0.01 (**). Group size estimations were based on a power calculation to minimally yield an 80% chance to detect a significant difference of *P* < 0.05 between groups.

## Supplementary Material

Supplementary File

Supplementary File

Supplementary File

Supplementary File

Supplementary File

Supplementary File

## Data Availability

All study data are included in the article and/or supporting information.

## Change History

September 9, 2022: The article license has been updated.
