# Supplementary material for: An immune-sympathetic neuron communication axis guides adipose tissue browning in cancer-associated cachexia
Source: Proc Natl Acad Sci U S A. 2022 Feb 24;119(9):e2112840119. doi: 10.1073/pnas.2112840119 (PMC8892347; doi:10.1073/pnas.2112840119)
Supplement: Supplementary File [file pnas.2112840119.sapp.pdf]

Supplemental Information

Supplemental Methods

Generation of DBHΔper mice.

DBH$^{flox/flox}$ mice were generated by micro-injecting EUCOMM targeted ES cells (Dbh$^{tm1a(EUCOMM)Wtsi}$, MGI: 5014701, KO first allele, clone EPD0800_2_B07) into C57Bl/6J-Albino blastocysts. After germline transmission by one chimeric mouse, the "conditional ready" Dbh^{tm1c} (floxed) allele was generated by *in vivo* deletion of the FRT-lacz-neo cassette by crossing mice with pCAG-Flpe mice (MGI:2448985). We generated inducible peripheral DBH-knockout mice by crossing DBH$^{flox/flox}$ mice with tamoxifen-inducible Rosa26CreERT2 mice (MGI: 3764519, C57Bl/6J background, a kind gift from Dr. Anton Berns, Amsterdam, Netherlands. DBH$^{flox/flox}$ (WT littermates) and DBH$^{flox/flox}$; Rosa26^{CreERT2} mice (DBHΔper mice) exhibited similar peripheral DBH protein expression before tamoxifen administration. Tamoxifen was administered to male 8-12 weeks old WT or DBHΔper, resulting in decreased DBH expression in peripheral tissues but not in the brain.

Isolation and cultivation of sympathetic neurons.

Murine pups were anesthetized and sacrificed by decapitation. Superior cervical ganglions (SCGs) were excised under a stereoscopic microscope. Excess tissues and debris were removed using fine forceps. Intact SCGs were stored in 1 ml of L15 Leibovitz media (#11415064, ThermoFisher, USA) and incubated with 4 mg/ml collagenase type 2 and 5 mg/ml dispase II (#4942078001, Roche Diagnostics, Switzerland) at 37 °C for 60 min and agitated every 10 min. After the incubation, the neuron suspension was centrifuged at 300 g and RT for 5 min and washed 3 times with PBS. After resuspension, dissociated neurons were filtered through a sterile 70 µm cell strainer into a 50 ml conical tube to remove any remaining cell aggregates. The dissociated neuron suspension was centrifuged at 300 g for 5 min and resuspended in prewarmed L15 Leibovitz full media (with 10 % FBS, 100 IU/l penicillin, and 0.1 mg/l streptomycin). Live cell number was counted using a hemocytometer (TC20, Bio-Rad, CA, USA) and 0.4 % trypan blue dye. 10,000 live cells were plated with 400 µl L15 Leibovitz full media on 0.01 % poly-D-lysine (#P6407-5MG, Sigma-Aldrich) and 3 µg/ml laminin (#23017-

015, Thermo Fisher Scientific, USA) coated cover glass II (#94.6190.802, SARSTEDT, Germany) in wells of 8-well culture chambers. SCG neurons were allowed to adhere to the bottom of the chambers for 2 h before the medium was changed to 300 µl pre-warmed L15 Leibovitz full media containing B-27 plus supplement (A3653401, Thermo Fisher Scientific, USA) and maintained at 37 °C in a humidified incubator with 5 % CO_2.

Antibodies used for Western blotting analyses

GAPDH (#2118S, 1:50000, Cell signaling, USA), UCP-1 (#14670S, 1:1000, Cell signaling), TH (#MAB318, 1:1000, Millipore, USA), DBH (#ab96615, 1:1000, Abcam, UK), MAO-A (#sc-20156, 1:1000, Santa Cruz, USA), P-HSL (#4126, 1:1000, Cell signaling), HSL (#4107S, 1:1000, Cell signaling), P-STAT3 (#9145S, 1:1000, Cell signaling), STAT3 (#4904S, 1:1000, Cell signaling), FABP4 (#ab81605, 1:1000, Abcam), F4/80 (#ab74383, 1:1000, Abcam), P-ERK (#9102, 1:1000, Cell signaling), ERK (#9101, 1:1000, Cell signaling), P-IRE1 (#ab48187, 1:1000, Abcam), IRE1 (#3294, 1:1000, Cell signaling).

HRP-linked Anti-rabbit IgG antibody (#A120-201P, 1:8000, Bethyl, USA) and HRP-linked Anti-mouse IgG antibody (#NA 931V, 1:8000, GE healthcare, USA) were used as secondary antibodies.

Volume fluorescence imaging of adipose tissue

Adipose tissue preparation, immunolabeling, clearing, and imaging was performed as described (1). Mice were anesthetized and perfused with PBS containing 10 µg/ml heparin (#B01AB01, Gilvasan, Austria). Adipose tissue was dissected and fixed in PBS/1 % PFA/10 % sucrose at 4 °C overnight. Tissues were dehydrated in 20 % methanol for 30 min, 40 % methanol for 30 min, 60 % methanol for 30 min, 80 % methanol for 30 min, and 100 % methanol for 30 min twice. Thereafter tissues were bleached with 5 % H_2O_2 containing 10 mmol/l EDTA (pH 8.0) for 48 h and rehydrated in 80 % methanol for 30 min, 60 % methanol for 30 min, 40 % methanol for 30 min, 20 % methanol for 30 min, and 0.2 % Triton X-100 for 1 h. Tissues were permeabilized in 0.2 % Triton X-100/20 % DMSO/0.3 mol/l glycine for 24 h and blocked in 0.2 % Triton X-100/ 10 % DMSO/5 % donkey serum for 24 h. Tissues were

(1) Y. Cao, H. Wang, W. Zeng, Whole-tissue 3D imaging reveals intra-adipose sympathetic plasticity regulated by NGF-TrkA signal in cold-induced beiging. *Protein Cell* **9**, 527–539 (2018).

stained with TH (#AB152, 1:1000, Millipore, USA) or GAP-43 (#8945S, 1:1000, Cell signaling, USA) antibodies in 0.2 % Tween-20/10 μg/ml heparin/5 % DMSO/5 % donkey serum for 72 h and with Alexa dye-conjugated secondary antibodies (1:500 in PBS/0.2 % Tween-20/10 μg/ml heparin/5 % donkey serum) for 72 h. Immunolabeled iWAT was embedded in 1 % agarose-blocks and dehydrated in 20 % methanol for 1 h, 40 % methanol for 1 h, 60 % methanol for 1 h, 80 % methanol for 1 h, and 100 % methanol for 1 h. Then, tissue blocks were incubated with dichloromethane (#270997, Sigma-Aldrich, USA)/methanol (2 volumes/1 volume) for 3 h, and with 100 % dichloromethane for 15 min. Tissue blocks were finally cleared using 100 % dibenzyl ether (#33630, Sigma-Aldrich, USA) for 1 h twice to be ready for volume fluorescence imaging. Optically cleared iWAT was imaged on the LaVisionBiotec Ultramicroscope II equipped with six fixed lightsheet generating lenses, the sCMOS camera (Andor Neo), and the 2×/NA0.5 objective (MVPLAPO) covered with the 6-mm working distance dipping cap using the Version v144 of the Inspector Microscope Controller software supported by LaVisionBiotec. For imaging at 1.26× effective magnification (0.63× zoom) tissue blocks were scanned by three combined lightsheets with a step-size of 4 or 5 μm through each tissue block. For imaging at 12.6× effective magnification (6.3× zoom), tissue blocks were scanned by a single lightsheet (middle position) with a step size of 1 μm through each tissue block. Image stacks were acquired by the continuous lightsheet scanning method without the contrast-blending algorithm.

Image stacks were reconstructed using Imaris (http://www.bitplane.com/imaris/imaris). Movies of the image stacks were generated with a frame rate of 15 fps. 3D projections of the image stacks were generated with the orthogonal perspective for the representative images shown. In each region, small cubic segments (at least 5 cubes per region) were randomly isolated using the surface tool of Imaris. TH or GAP-43 signal in each cube was reconstructed using the Filament Tracer tool of Imaris. Regional volumes (mm^3) and total neurite length (mm) of the cubes were automatically calculated by the surface and Filament Tracer tools of Imaris, respectively. Neurite density in each cube was calculated as the ratio of total neurite length by regional volume.

Supplemental Table 1: List of primer sequences used for qRT-PCR

Gene name	Symbol	Primer sequence
Ucp-1	*Ucp-1*_fwd	5'-ACTGCCACACCTCCAGTCATT-3'
	*Ucp-1*_rev	5'-CTTTGCCTCACTCAGGATTGG-3'
β-Actin	*β-Actin*_fwd	5’-AGCCATGTACGTAGCCATCCA-3’
	*β-Actin*_rev	5’-TCTCCGGAGTCCATCACAATG-3’
Pgc-1α	*Pgc-1α*_fwd	5'-CCCTGCCATTGTTAAGACC-3'
	*Pgc-1α*_rev	5'-TGCTGCTGTTCCTGTTTTC-3'
Dio2	*Dio2*_fwd	5'-CAGTGTGGTGCACGTCTCCAATC-3'
	*Dio2*_rev	5'-TGAACCAAAGTTGACCACCAG-3'
Arg1	*Arg1*_fwd	5'-CTCCAAGCCAAAGTCCTTAGAG-3'
	*Arg1*_rev	5'-AGGAGCTGTCATTAGGGACATC-3'
IL10	*IL-10*_fwd	5'-GCTCTTACTGACTGGCATGAG-3'
	*IL-10*_rev	5'-CGCAGCTCTAGGAGCATGTG-3'
iNos	*iNos*_fwd	5'-CACCAAGCTGAACTTGAGCG-3'
	*iNos*_rev	5'-CGTGGCTTTGGGCTCCTC-3'
Tgfβ1	*Tgfβ1*_fwd	5'-CCTGTCCAAACTAAGGC-3'
	*Tgfβ1*_rev	5'-GGTTTTCTCATAGATGGCG-3'
Tgfβ2	*Tgfβ2* fwd	5'-CAGGAGTGGCTTCACCACAAAG-3'
	*Tgfβ2*_rev	5'-TGGCATATGTAGAGGTGCCATCA-3'
Tgfβ3	*Tgfβ3*_fwd	5'-CACCGGAGAGCCCTGGATA-3'
	*Tgfβ3*_rev	5'-TGTACAGCTGCCGCACACA-3'
Ngf	*Ngf*_fwd	5'-TGA TCG GCG TAC AGG CAG A-3'
	*Ngf*_rev	5'-GAG GGC TGT GTC AAG GGA AT-3'
Bdnf	*Bdnf*_fwd	5'- GCT TTG CGG ATA TTG CGA AGG GTT-3'
	*Bdnf*_rev	5'- CAC CTG GTG GAA CAT TGT GGC TTT-3'
Gap-43	*Gap-43*_fwd	5'-CAG GAA AGA TCC CAA GTC CA-3'
	*Gap-43*_rev	5'-GAA CGG AAC ATT GCA CAC AC-3'

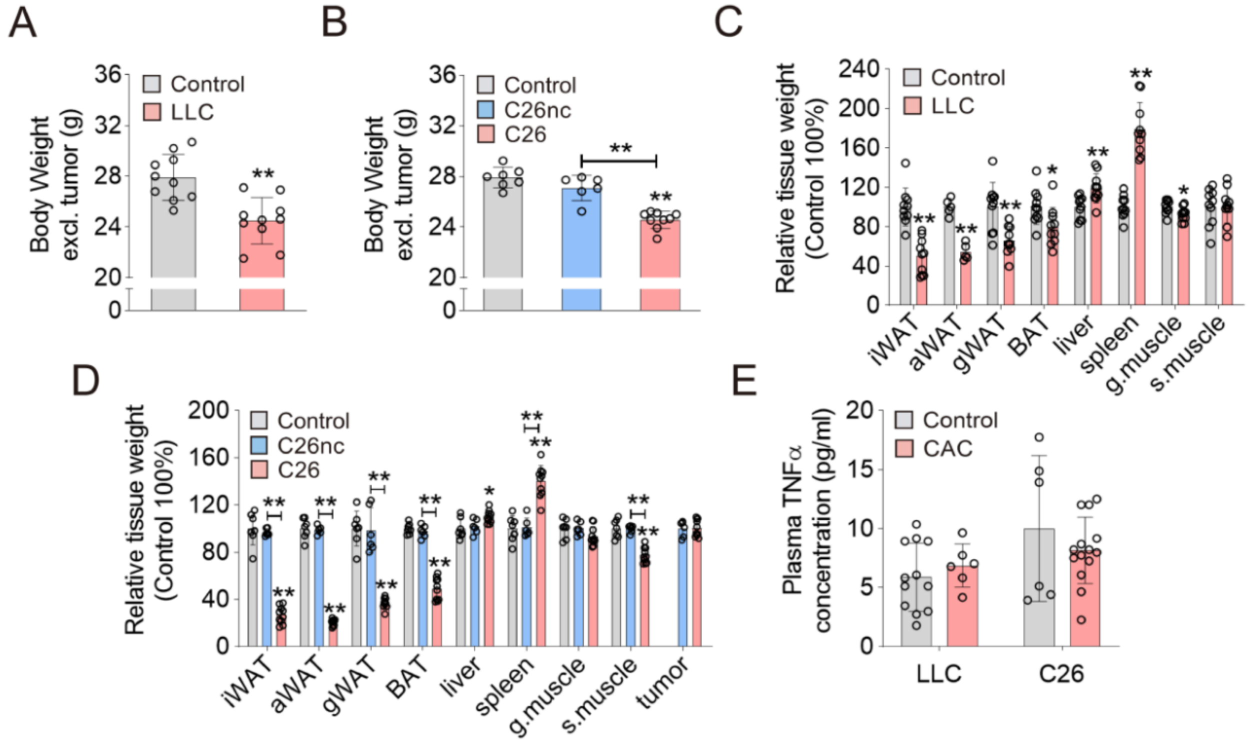

Supplemental Figures and Legends

Figure S1: LLC and C26 tumor-bearing mice exhibit body weight reduction, adipose tissue loss, and muscle tissue atrophy, but no increase in plasma TNFα concentrations. Mice bearing LLC, C26, and C26nc tumors were sacrificed 14-16 days after tumor cell inoculation (10-12 weeks old). **(A,B)** Net body weights of LLC **(A)**, C26, and C26nc **(B)** tumor-bearing mice (n= 6-10 per group, results of two independent experiments were combined). **(C,D)** Tissue weights of LLC **(C)**, C26, and C26nc **(D)** tumor-bearing mice relative to tissue weights of control animals (n= 10 per group, results of two independent experiments were combined). **(E)** TNFα plasma concentration was determined in LLC, C26, and C26nc tumor-bearing mice using ELISA (n= 6-14 per group). Data are presented as means ± standard deviation. Significance was determined by unpaired two-tailed Student's t-test **(A,C,E)**, or a one-way ANOVA followed by Tukey's *post hoc* analysis **(B,D)** (*p≤0.05, **p ≤ 0.01).

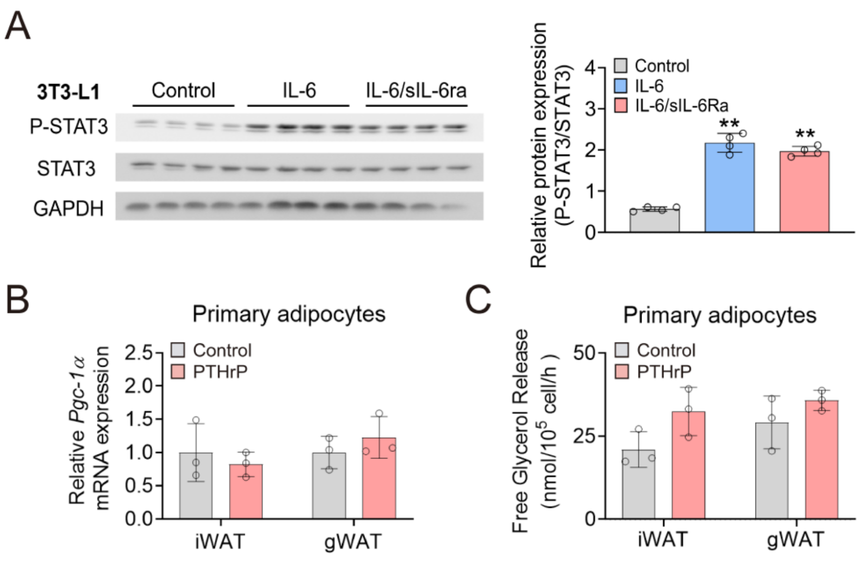

Figure S2: Functional IL-6 signaling in 3T3-L1 adipocytes. PTHrP does not increase primary adipocytes Pgc1α mRNA expression or glycerol release. (A) Western blotting analysis to detect P-STAT3 and STAT3 protein contents in differentiated 3T3-L1 adipocytes that were incubated in the presence and absence of 20 ng/ml IL-6, 20 ng/ml IL-6/37.14 ng/ml sIL-6ra, or vehicle for 8 h. GAPDH was used as loading control. The protein contents of P-STAT3 and STAT3 were quantified by densitometric analysis. **(B)** qRT-PCR analysis was used to detect mRNA levels of *Pgc-1α* in primary adipocytes isolated from iWAT and gWAT that were treated with 10 ng/ml PTHrP or vehicle for 8 h (n=3 per condition). **(C)** Glycerol release from primary adipocytes treated with 40 ng/ml PTHrP or vehicle and 2 % FA-free BSA for 8 h. Data are presented as means ± standard deviation. Significance was determined by unpaired two-tailed Student's t-test (**B,C)**, or a one-way ANOVA followed by Tukey's *post hoc* analysis **(A)** (*p≤0.05, **p ≤ 0.01).

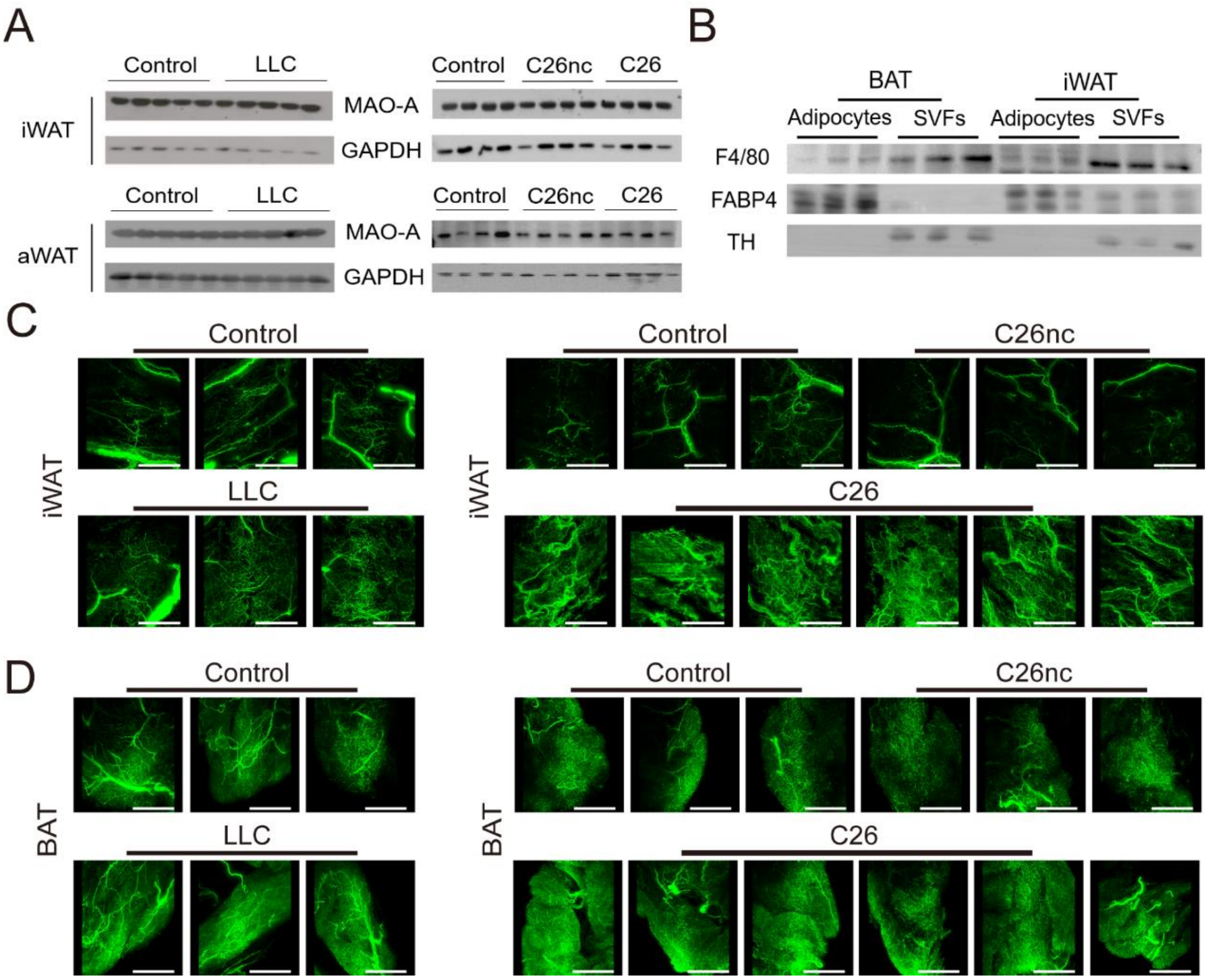

Figure S3: Monoamine oxidase A expression is unchanged in adipose tissue of cachectic mice. TH is exclusively expressed in the stromal vascular fraction of adipose tissue. (A,C,D) Mice bearing LLC, C26, and C26nc tumors were sacrificed 14-16 days after tumor cell inoculation (10-12 weeks old). **(A)** Western blotting analysis to detect MAO-A protein expression in iWAT and aWAT of LLC, C26, and C26nc tumor-bearing or control mice. GAPDH was used as loading control. **(B)** Western blotting analysis to detect TH protein expression in different fractions of iWAT and BAT. FABP4 was used as marker for adipocytes and F4/80 as marker for stromal vascular cells (SVFs). **(C,D)** 3D projections of iWAT **(C)** and BAT **(D)** sections from LLC, C26, and C26nc tumor-bearing mice immunolabeled for TH, and imaged at 12.6×magnification on a light sheet microscope (n=3-6 per group). Scale bars, 500 µm. Data are presented as means ± standard deviation. Significance was determined by unpaired two-tailed Student's t-test or a one-way ANOVA followed by Tukey's *post hoc* analysis (*p≤0.05, **p ≤ 0.01).

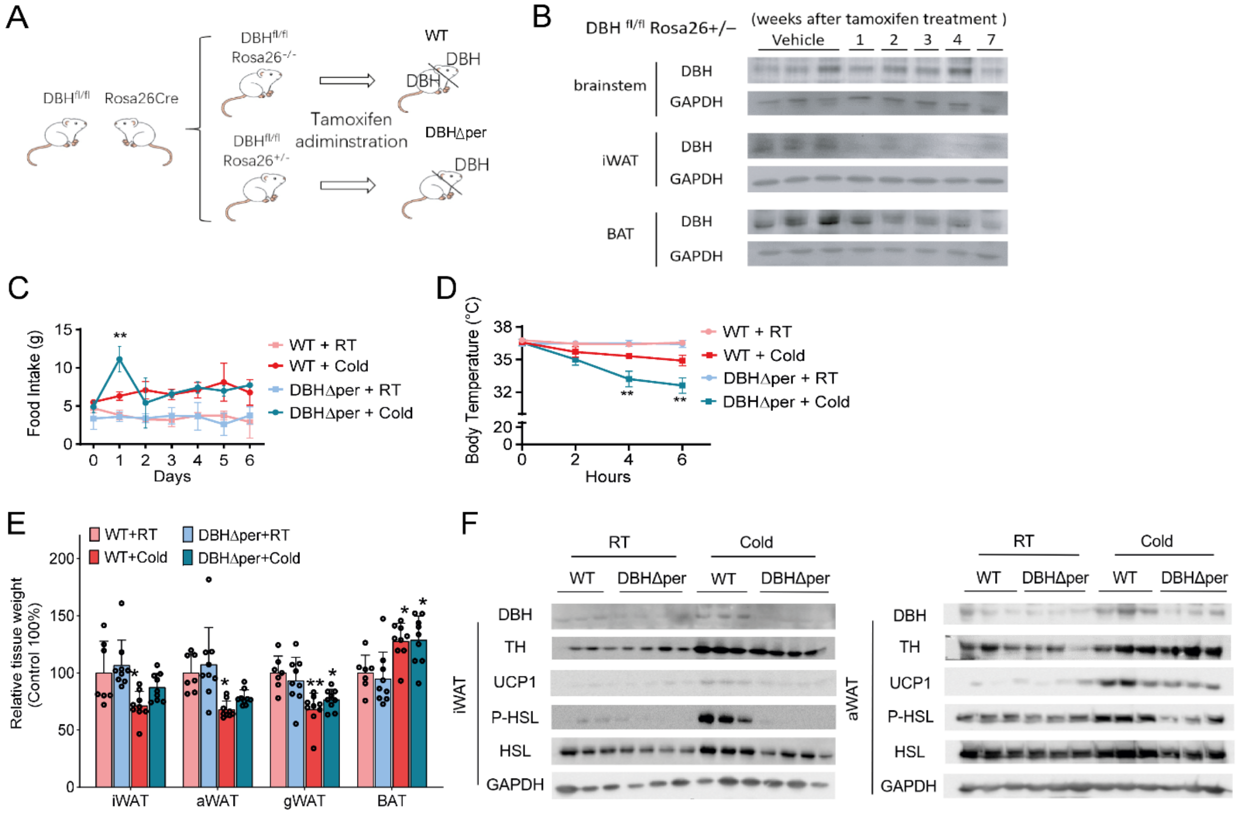

Figure S4: Peripheral DBH deficiency impairs thermoregulation, lipolysis, and adipose tissue browning of cold exposed mice. (A) Schematic overview of the strategy to generate inducible peripheral DBH knockout (DBHΔper) mice. **(B)** Western blotting analysis to detect DBH protein expression in brainstem, iWAT, and BAT from DBHflox/flox,Rosa26CreERT2+/- mice at different timepoints after vehicle or tamoxifen treatment. **(C-F)** WT and DBHΔper mice (10-12 weeks old) were single housed at 21 °C or 4 °C for 1 week with ad libitum access to water and chow diet. **(C)** Food intake of WT and DBHΔper mice at 4 °C or 21 °C over a period of 7 days (n= 3-5 per group). **(D)** Body temperature of WT and DBHΔper mice during fasting at 4 °C or 21 °C for 6 h (n= 4-5 per group). **(E)** Adipose tissue weights of DBHΔper and WT mice relative to tissue weights of WT animals at 21 °C (n= 10 per group, results of two independent experiments were combined). **(F)** Western blotting analysis to detect DBH, TH, UCP-1, P-HSL (Ser660), and HSL protein contents in iWAT and aWAT**.** GAPDH was used as loading control. Data are presented as means ± standard deviation. Significance was determined by two-way ANOVA followed by Tukey's *post hoc* analysis (*p≤0.05, **p ≤ 0.01).

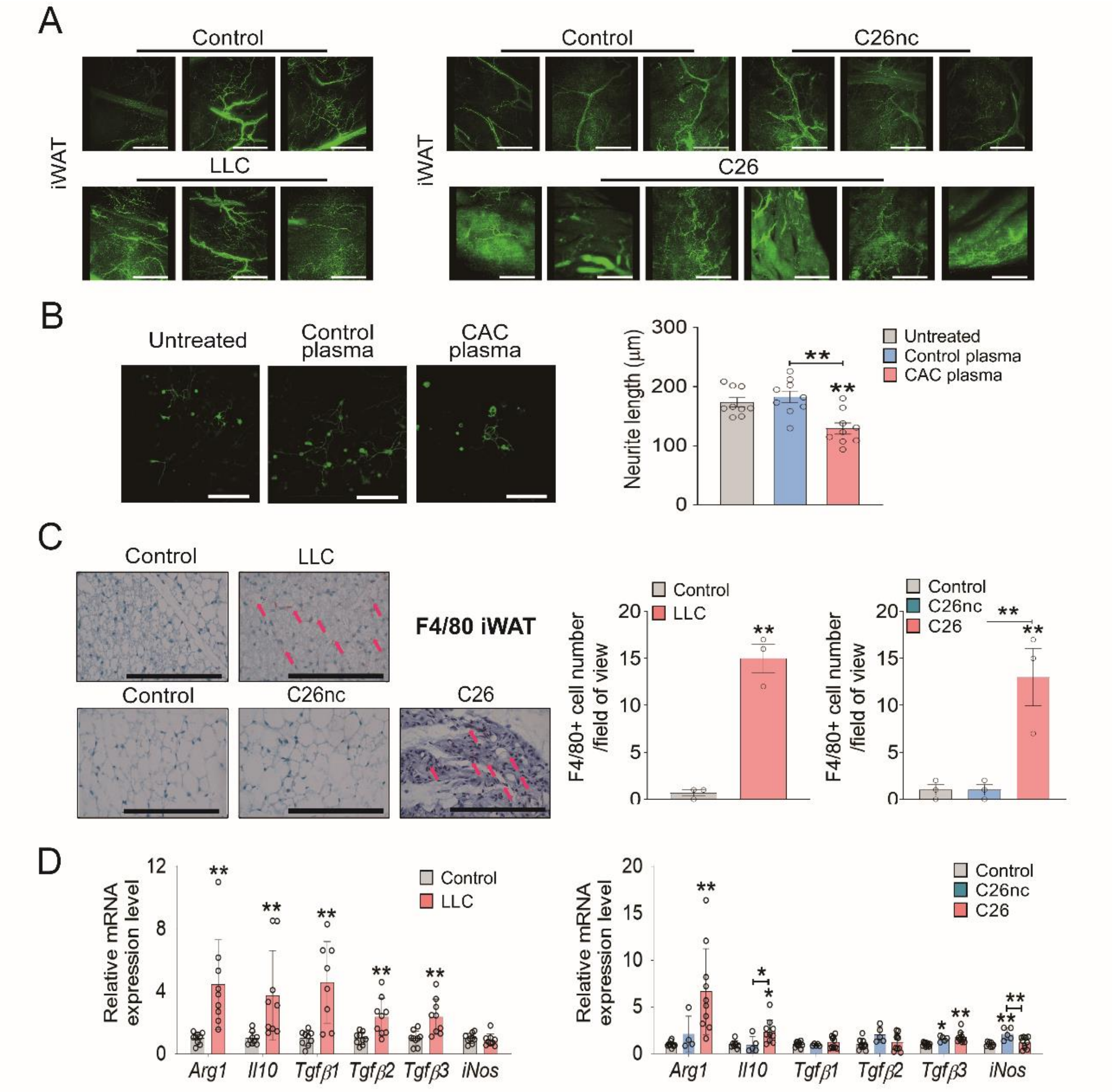

Figure S5: Adipose tissues of cachexigenic tumor-bearing mice exhibits increased neurite outgrowth, macrophage infiltration, and type 2 immune cell activation. (A,C,D) Mice bearing LLC, C26, or C26nc tumors were sacrificed 14-16 days after tumor cell inoculation (10-12 weeks old). **(A)** Representative 3D projections of iWAT sections from LLC, C26, and C26nc tumor-bearing mice immunolabeled for GAP-43, and imaged at 12.6× magnification on a light sheet microscope (n= 3-6 per group). Scale bars, 500 μm. **(B)** Representative images of cultured primary sympathetic neurons grown in the presence or absence of 2 % plasma derived from control- or LLC tumor-bearing mice (CAC), immunolabeled for TH, and imaged by confocal fluorescence microscopy. Scale bars, 250 μm. Neurite length was determined using the Neuron J plugin of Image J (n= 9 per condition). **(C)** Representative sections of iWAT from control, LLC, C26, and C26nc tumor-bearing mice, immunolabeled for F4/80, scale bars, 200 μm. Quantification of F4/80 positive cells was performed on sections of 3 individual mice

per group. **(D)** qRT-PCR analysis was used to detect mRNA levels of type 1 and type 2 immune marker genes in iWAT. *β-actin* was used as housekeeping gene (n= 5-10 per group, results from 2 independent experiments were combined). Data are presented as means ± standard deviation. Significance was determined by two-tailed student's t-test **(C,D)**, or a one-way ANOVA followed by Tukey's *post hoc* analysis **(B-D)** (*p≤0.05, **p ≤ 0.01).

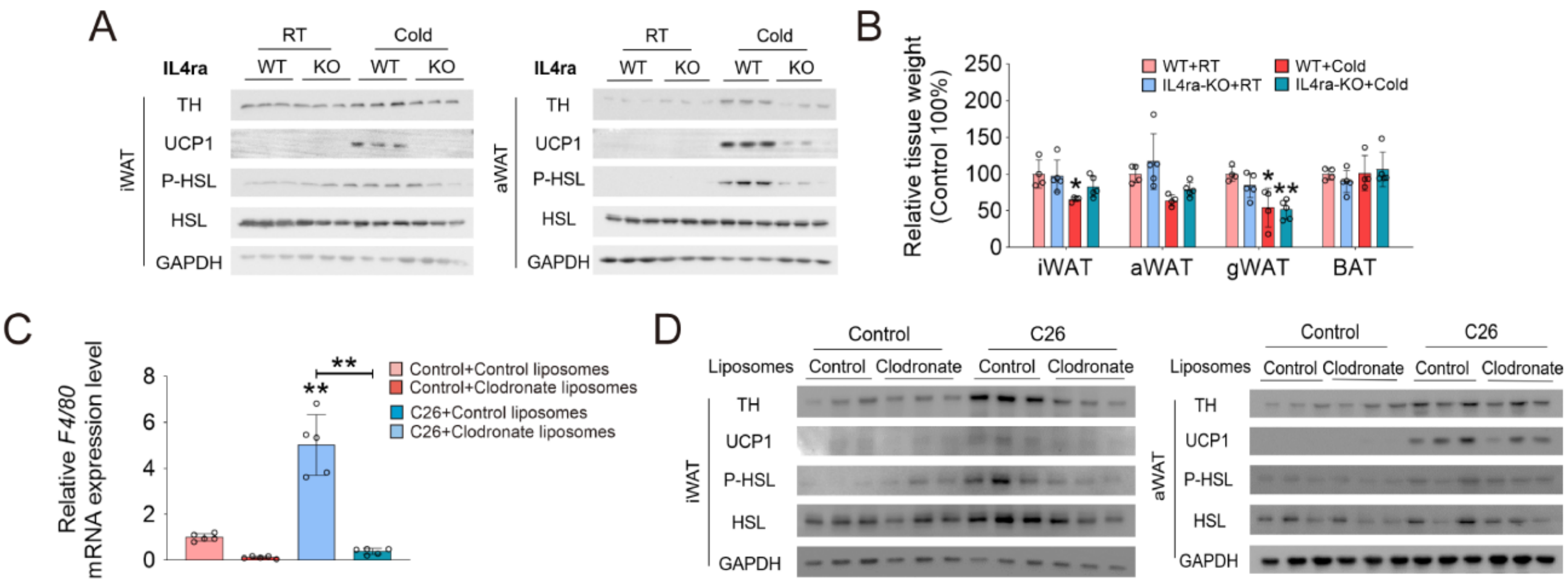

Figure S6: IL-4ra deficiency or local clodronate injection impairs browning and HSL activation in WAT of cold exposed- or tumor-bearing mice, respectively. (A,B) WT and IL4ra-KO mice (10-12 weeks old) were single housed at 21 °C or 4 °C for 1 week with ad libitum access to water and chow diet. **(A)** Western blotting analysis to detect TH, UCP-1, P-HSL (Ser660), and HSL protein contents in iWAT and aWAT. GAPDH was used as loading control. **(B)** Adipose tissue weights relative to tissue weights of WT animals at 21 °C (n= 4-5 per group). **(C,D)** C26 tumor-bearing mice and control mice were subcutaneously injected with control/clodronate liposomes every two days and sacrificed 14-16 days after tumor cell inoculation (10-12 weeks old). **(C)** qRT-PCR analysis was used to detect mRNA level of *F4/80* in iWAT (n= 4 per group). **(D)** Western blotting analysis to detect TH, UCP-1, P-HSL (Ser660), and HSL protein contents in iWAT and aWAT; GAPDH was used as loading control. Data are presented as means ± standard deviation. Significance was determined by two-way ANOVA followed by Tukey's *post hoc* analysis (*$p \leq 0.05$, **$p \leq 0.01$).

Supplemental Videos 1-5: Increased TH expression in neurites of the sympathetic nervous system in adipose tissues of LLC and C26 tumor-bearing mice. (1-2) 3D projections of iWAT from control (1) and cachectic LLC tumor-bearing (2) mice and (3-5) from control (3), C26nc (4), and C26 (5) tumor-bearing mice immunolabeled for TH, and imaged at 2.6×magnification on a light sheet microscope. Scale bars, 500 µm.
